# The trend of AIDS in China: A prediction and comparative analysis with G20 countries based on the Global Burden of Disease Study 2019

**DOI:** 10.7189/jogh.14.04029

**Published:** 2024-03-01

**Authors:** Mimi Zhai, Xianyang Lei, Yunxia Li, Li Li, Qin Jiang, Yamin Li, Sushun Liu

**Affiliations:** 1 Clinical Nursing Teaching and Research Section, the Second Xiangya Hospital, Central South University, Changsha, Hunan, China; 2 Xiangya Nursing School, Central South University, Changsha, Hunan, China; 3 Xiangya School of Public Health, Central South University, Changsha, Hunan, China; 4 Department of Urology, the First Affiliated Hospital of Xinjiang Medical University, Urumqi, Xinjiang, China; 5 Department of General Surgery, the Second Xiangya Hospital, Central South University, Changsha, Hunan, China

## Abstract

**Background:**

In China, AIDS has become the most severe notifiable infectious disease. The study aimed to analyse and predict the trend of AIDS in China and compared with Group of Twenty (G20) countries.

**Methods:**

We utilised incidence, mortality or disability-adjusted life years (DALY), age-standardised rates (ASR), average annual percentage changes (AAPC) to estimate the trend via GBD 2019. The Joinpoint regression analysis was applied to identify the most significant years of change. We explored the relationship between AAPC and social development index (SDI) or health care access and quality (HAQ), and predicted trends for the next 20 years.

**Results:**

The DALY in G20 increase of 340.42%, and 794.50% in China. The age-standardised DALY rate (ASDR) in G20 was 309.49 (95% uncertainty interval (UI) = 284.69, 350.58) in 2019, with an AAPC of 4.30. Among G20, the United States had the highest DALY in 1990, but it experienced a significant decline. In China, the ASDR was 98.15 (95% UI = 78.78, 119.58) with the 5th AAPC ranking. In term of gender, the incidence, mortality, DALY, and ASR of them in China and G20 were all higher in males. Furthermore, the gender gap in China had been widening. The most significant periods of ASDR increase in China were 1990–1995 and 2013–2016, and 1990–1994 in G20. The prediction for DALY indicated that high SDI countries were expected to exhibit a stable or declining trend, while low SDI countries showed an upward trend. China demonstrated a 57.66% increase in 2040 compared to 2019.

**Conclusions:**

AIDS continues to be a significant burden. In China, the ASIR exhibited a decline trend in certain age groups, while the ASMR and ASDR continued to increase, with a widening gender disparity. In addition, according to our predict results, some countries could not achieve the 2030 Agenda for Sustainable Development set by the UNAIDS. Therefore, it is necessary to establish more effective and targeted measures, as well as actively explore new treatment approaches.

AIDS is a severe immune system disease caused by HIV, and was first identified over forty years ago. Currently, AIDS still maintains a high incidence and mortality rate, continuing to affect millions of people worldwide. In addition, AIDS generates billions of dollars in direct health care costs and indirect socio-economic costs each year, imposing a significant burden on society [1].

In China, AIDS is the most severe notifiable infectious disease. However, current AIDS research in China mainly focuses on cross-sectional studies at the provincial level, which fails to comprehensively depict dynamic changes and limit the understanding of AIDS trends. Moreover, studies on AIDS mainly focused on incidence and mortality, with limited researches on change in epidemic trend of disability-adjusted life years (DALY) [2–6]. Notably, DALY comprehensively assesses the severity of disease in terms of both mortality and disability, providing an objective measure of disease burden. By evaluating the trend of DALY, we can obtain a more precise understanding of the threat that AIDS poses to people's health and lives.

To gain a more comprehensive understanding of the epidemiological trends of AIDS in China, this study not only analyses DALY trends across different countries, genders, and age groups but also introduces the latest assessment indicators, namely health care access and quality (HAQ). Furthermore, we compared China to Group of Twenty (G20) countries, which represent the most influential and diverse economies globally. Through this comparison, we aimed to explore China's strengths and weaknesses in controlling the spread of AIDS and draw insights from the experiences of other nations. Lastly, we examined the relationship between average annual percentage changes (AAPC) and social development index (SDI) or HAQ, and forecast the future trends of AIDS in China and G20 countries. This study is dedicated to addressing two crucial scientific questions. First, it aims to analyse the epidemiological trends of AIDS in China over the past three decades. Second, it seeks to compare China's AIDS situation with that of G20 countries, identifying China's strengths and weaknesses, and drawing lessons from the experiences of these countries. Through this research, we aspire to provide a scientific foundation for formulating more precise prevention and control strategies. This will contribute to alleviating the heavy burden of AIDS on Chinese society and the economy.

## METHODS

### Data sources

This research is an observational study. We utilised the data from GBD 2019, which provides a comprehensive assessment of various health indicators such as incidence, mortality, years of life lost (YLL), years lived with disability (YLD), and more. The GBD study covers 369 diseases and injuries across 204 countries and regions, spanning the period from 1990 to 2019. It is important to note that the data used in our study does not include any information that could potentially compromise patients' privacy or reveal individually identifiable details. Furthermore, we adhered to the guidelines outlined in the Creative Commons Attribution-NonCommercial-NoDerivatives 4.0 International License, as well as Section 7 of the University of Washington's Website Terms and Conditions of Use, ensuring that the data was used in accordance with the appropriate legal and ethical regulations.

### Socio-demographic index

We used the socio-demographic index to measure the level of development in regions based on the composite factors of total fertility rate, lagged income distribution per capita, and average years of education for individuals aged 15 and above. The socio-demographic index ranges from 0 to 1 and categorises regions into low, low-middle, middle, high-middle, and high socio-demographic regions based on their scores.

### Healthcare access and quality

We employed the HAQ in our study to assess the level of health care access and quality in different regions and countries [7]. It determines the health care status of countries and regions by assessing causes of death that should not occur when effective medical care is available, and provides a comprehensive measurement of health care performance by considering several dimensions, including availability, effectiveness, and quality of health care services [8]. The HAQ ranges from 0 to 100, with 0 representing the worst and 100 representing the best health care outcomes.

### Trend prediction

We used an ARIMA model to predict the incidence, mortality, DALY, and mortality-incidence ratio (MIR). The xtarima command in STATA 17.0 was employed to identify the optimal ARIMA (p, d, q) model to analyse the trend of these indicators, as well as predict their values from 2020 to 2040. For the prediction, the corresponding population data from United Nations Department of Economics and Social Affairs Population Division, covering the years 1990 to 2040, genders and ages, was utilised as reported in our previous study [9].

### Statistical analysis

We used incidence, mortality, DALY and MIR to describe the prevalence and severity of AIDS epidemic at specific time points. DALY served as an indicator of health loss, calculated as the sum of YLL and YLD, as reported in our previous study [9]. MIR served as an index to compare the disease burden by standardising mortality to incidence [10]. The MIR provides insights into the outcome or impact of a disease by comparing the number of deaths it causes to the number of new cases that arise [10]. In addition, we used ASR and AAPC in our study to describe the trend of AIDS from 1990 to 2019, as our previous study [9,11].

We employed joinpoint regression analysis to examine the long-term trends in AIDS in China and G20 countries by joint command line. In this model, each joinpoint reflected a statistically significant change, and each trend was described by APC [9,12]. The correlations between AAPC and SDI or HAQ were evaluated using Pearson correlation analyses. All statistical analysis and data visualisations were performed using Stata 17.0. *P* < 0.05 was considered to be statistically significant.

## RESULTS

### Overview of AIDS trends

**Figure 1:**
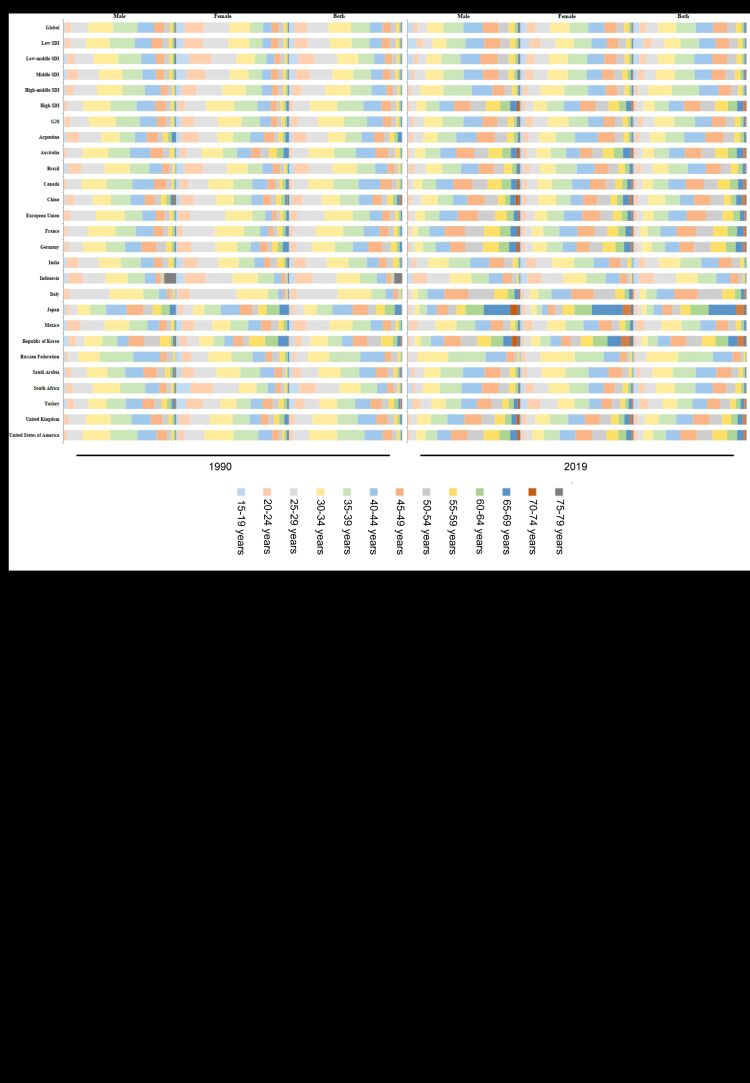
The distribution of disability-adjusted life years (DALY) across different age groups and genders in Group of Twenty (G20) countries and social development index (SDI) regions.

**Figure 2:**
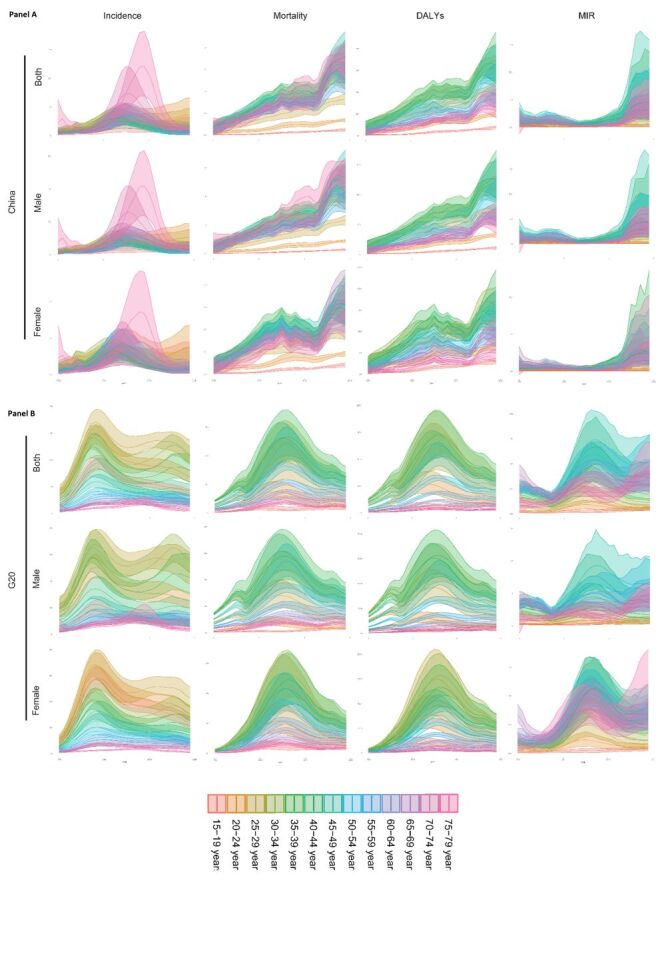
The trend of age-standardised incidence rate (ASIR), age-standardised mortality rate (ASMR), age-standardised DALY rate (ASDR), and age-standardised rate (ASR) of mortality-incidence ratio (MIR) for AIDS in China and the Group of Twenty (G20) countries across in different age groups. **Panel A.** The trend of ASIR, ASMR, ASDR and ASR of MIR among males, females, and both sexes in different age groups in China from 1990 to 2019. **Panel B.** The trend of ASIR, ASMR, ASDR and ASR of MIR in G20.

**Figure 3:**
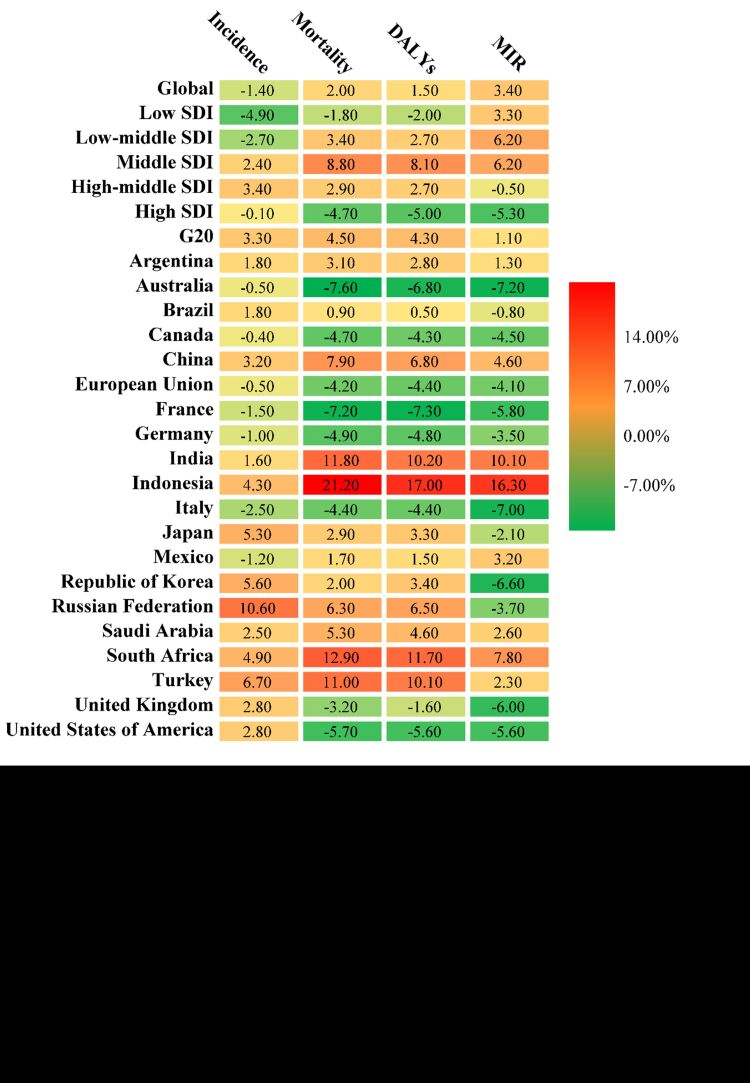
The overall average annual percent change (AAPC) of age-standardised incidence rate (ASIR), age-standardised mortality rate (ASMR), age-standardised DALY rate (ASDR) and age-standardised rate (ASR) of mortality-incidence ratio (MIR) in the Group of Twenty (G20) countries and social development index (SDI) regions.

In 2019, the DALY in G20 countries was 15.05 million, with the ASDR of 309.49 (95% uncertainty interval (UI) = 284.69, 350.58). The DALY increased by 340.42% from 1990 to 2019, and its AAPC was 4.30 (95% UI = 3.60, 5.00) (**Table 1**, **Figure 1**, **Figure 2**, and **Figure 3**). The lowest ASDR in 1990 was found in Indonesia with a value of 0.62 (95% UI = 0.00, 1.08), but it increased to 161.64 (95% UI = 130.76, 201.89) in 2019, and became the country with the largest increase in ASDR. The AAPC of ASDR in Indonesia was 17.00 (95% UI = 13.50, 20.50) (**Figure 3**). In addition, South Africa consistently suffered with a high ASDR during this period. The AAPC of South Africa was 11.70 (95% UI = 10.30, 13.10), which was only lower than Indonesia (**Table 1**, **Figure 1**). Although the United States obtained the highest ASDR in 1990, it showed a third largest decline in ASDR with a value of 126.63 (95% UI = 105.65, 155.02) in 2019. The AAPC was −5.60 (95% UI = −6.50, −4.60), following France and Australia (**Table 1**, **Figure 1** and **Figure 3**). It was worth noting that China’s ASDR was 13.18 (95% UI = 4.52, 18.00) in 1990, and increase to 98.15 (95% UI = 78.78, 119.58) in 2019. China ranked the 5th of the change of ASDR, with the AAPC of 6.80 (95% UI = 5.80, 7.90) (**Table 1**, **Figure 1**, **Figure 2**, panel A, **Figure 3**).

**Table 1:** The DALY cases, ASDR, and temporal trend of AIDS from 1990 to 2019 in China and G20

Characteristics	1990			2019			1990–2019
	**DALY cases**	**ASDR per 100 000**	**DALY cases**		**ASDR per 100 000**	**AAPC**	
	**No. ×10^3^ (95% UI)**	**No. (95% UI)**	**No. ×10^3^ (95% UI)**		**No. (95% UI)**	**No. (95% UI)**	
**China**							
**Total**	156.07 (53.48, 213.06)	13.18 (4.52, 18.00)	1396.03 (1120.50, 1700.88)		98.15 (78.78, 119.58)	6.80 (5.80, 7.90)	
**Male**	99.53 (32.54, 138.44)	16.31 (5.33, 22.69)	1033.35 (834.66, 1254.35)		142.57 (115.15, 173.06)	7.40 (6.40, 8.40)	
**Female**	56.54 (20.94, 75.53)	9.86 (3.65, 13.17)	362.67 (284.63, 446.36)		51.99 (40.81, 63.99)	5.60 (4.30, 7.00)	
**Age**							
15 to 19	2.59 (1.20, 3.88)	2.04 (0.95, 3.06)	14.63 (10.86, 17.11)		19.47 (14.46, 22.77)	7.90 (7.30, 8.60)	
20 to 24	10.49 (3.79, 16.03)	7.92 (2.87, 12.10)	41.57 (35.69, 45.81)		50.78 (43.59, 55.96)	6.50 (6.00, 7.00)	
25 to 29	19.96 (5.49, 31.33)	18.11 (4.98, 28.43)	118.86 (99.80, 138.74)		107.36 (90.14, 125.30)	6.20 (5.60, 6.90)	
30 to 34	18.15 (4.83, 28.60)	20.51 (5.46, 32.32)	195.27 (158.60, 238.71)		151.26 (122.85, 184.91)	6.80 (6.00, 7.50)	
35 to 39	19.14 (4.45, 30.94)	20.92 (4.86, 33.82)	173.92 (128.83, 239.97)		172.38 (127.69, 237.84)	7.10 (6.20, 8.10)	
40 to 44	13.50 (2.96, 21.52)	20.07 (4.41, 32.01)	164.24 (121.62, 210.87)		161.58 (119.65, 207.46)	6.50 (5.40, 7.50)	
45 to 49	9.22 (1.98, 14.25)	17.82 (3.83, 27.56)	190.09 (135.79, 257.41)		156.63 (111.88, 212.10)	7.10 (5.80, 8.50)	
50 to 54	6.19 (1.24, 9.14)	12.94 (2.59, 19.13)	158.65 (117.47, 207.60)		126.81 (93.90, 165.94)	8.20 (6.10, 10.40)	
55 to 59	5.16 (1.05, 7.48)	11.87 (2.41, 17.21)	81.09 (62.49, 101.38)		85.50 (65.89, 106.90)	6.50 (5.50, 7.40)	
60 to 64	4.24 (0.94, 6.12)	11.97 (2.66, 17.27)	61.91 (48.66, 76.46)		78.81 (61.95, 97.33)	6.10 (5.00, 7.10)	
65 to 69	2.63 (0.53, 3.85)	9.60 (1.95, 14.05)	65.55 (51.77, 81.15)		93.14 (73.55, 115.29)	7.60 (6.20, 9.10)	
70 to 74	1.17 (0.26, 1.77)	6.20 (1.39, 9.41)	37.38 (28.12, 47.72)		78.12 (58.76, 99.72)	8.70 (8.00, 9.50)	
75 to 79	1.47 (0.91, 1.90)	12.89 (7.99, 16.63)	17.11 (12.41, 22.45)		57.34 (41.58, 75.21)	5.50 (3.80, 7.20)	
**G20**							
**Total**	3417.64 (3261.97, 3577.60)	92.31 (88.10, 96.63)	15052.14 (13846.37, 17050.73)		309.49 (284.69, 350.58)	4.30 (3.60, 5.00)	
**Male**	2704.69 (2605.21, 2804.87)	144.60 (139.29, 149.96)	8213.52 (7468.94, 9378.93)		335.51 (305.10, 383.12)	3.00 (2.10, 3.80)	
**Female**	712.95 (657.37, 781.17)	38.91 (35.88, 42.64)	6838.62 (6021.75, 8134.60)		283.11 (249.29, 336.76)	7.10 (6.10, 8.10)	
**Age**							
15 to 19	49.86 (45.02, 57.68)	14.17 (12.79, 16.39)	446.88 (349.22, 578.71)		129.50 (101.20, 167.70)	7.90 (7.40, 8.50)	
20 to 24	185.48 (171.73, 201.91)	53.62 (49.65, 58.38)	728.69 (558.07, 965.06)		208.35 (159.56, 275.93)	4.80 (4.20, 5.50)	
25 to 29	525.70 (496.10, 559.54)	167.68 (158.24, 178.47)	1492.39 (1052.92, 2066.00)		398.48 (281.13, 551.63)	3.00 (1.90, 4.10)	
30 to 34	693.90 (665.69, 728.14)	251.41 (241.18, 263.81)	2417.22 (1960.46, 3041.54)		618.94 (501.98, 778.80)	3.10 (2.00, 4.30)	
35 to 39	615.40 (590.49, 645.41)	235.22 (225.69, 246.69)	2565.20 (2077.23, 3186.61)		727.62 (589.21, 903.88)	4.00 (3.00, 5.10)	
40 to 44	426.07 (408.85, 444.94)	197.73 (189.74, 206.48)	2127.65 (1913.48, 2386.59)		640.51 (576.04, 718.46)	4.20 (3.00, 5.40)	
45 to 49	231.43 (220.86, 242.90)	133.71 (127.60, 140.34)	1670.09 (1408.61, 1980.08)		498.48 (420.43, 591.00)	4.70 (3.70, 5.70)	
50 to 54	124.02 (117.20, 130.40)	77.89 (73.60, 81.89)	1160.30 (1014.81, 1363.27)		364.61 (318.89, 428.39)	5.50 (4.80, 6.30)	
55 to 59	76.56 (71.67, 81.22)	54.20 (50.73, 57.49)	676.51 (593.75, 794.09)		249.37 (218.87, 292.71)	5.50 (4.40, 6.70)	
60 to 64	46.11 (42.24, 49.09)	37.42 (34.28, 39.84)	394.03 (344.32, 463.98)		169.08 (147.75, 199.09)	5.40 (4.40, 6.40)	
65 to 69	26.77 (24.49, 28.62)	27.87 (25.49, 29.80)	238.18 (208.52, 278.73)		119.28 (104.43, 139.59)	5.20 (4.30, 6.10)	
70 to 74	4.48 (3.42, 5.33)	6.84 (5.23, 8.15)	104.67 (88.67, 125.27)		71.16 (60.28, 85.17)	8.40 (8.00, 8.80)	
75 to 79	3.47 (2.67, 4.20)	7.16 (5.51, 8.66)	45.89 (38.82, 55.18)		45.93 (38.85, 55.23)	6.60 (5.80, 7.40)	
**Regions**							
Argentina	31.56 (29.95, 33.56)	95.30 (90.44, 101.31)	102.49 (90.83, 121.18)		227.17 (201.33, 268.61)	2.80 (1.60, 4.00)	
Australia	19.55 (18.79, 20.40)	115.94 (111.43, 121.02)	4.29 (3.65, 5.21)		17.48 (14.87, 21.21)	−6.80 (−8.70, −4.80)	
Brazil	484.45 (472.34, 498.11)	325.49 (317.36, 334.67)	807.43 (773.53, 849.40)		372.66 (357.02, 392.04)	0.50 (0.00, 1.00)	
Canada	43.08 (40.77, 46.22)	158.04 (149.57, 169.56)	17.76 (14.70, 22.25)		48.64 (40.26, 60.94)	−4.30 (−5.90, −2.80)	
China	156.07 (53.48, 213.06)	13.18 (4.52, 18.00)	1396.03 (1120.50, 1700.88)		98.15 (78.78, 119.58)	6.80 (5.80, 7.90)	
European Union	573.51 (560.94, 586.99)	119.78 (117.15, 122.59)	211.06 (189.17, 239.35)		41.01 (36.75, 46.50)	−4.40 (−5.20, −3.50)	
France	158.50 (154.10, 163.45)	274.36 (266.75, 282.93)	26.24 (22.69, 30.92)		39.64 (34.28, 46.70)	-7.30 (-9.10–5.50)	
Germany	85.14 (82.28, 88.46)	106.50 (102.93, 110.65)	23.32 (20.50, 27.98)		27.46 (24.14, 32.95)	-4.80 (-5.70–3.90)	
India	99.27 (77.05, 137.20)	11.60 (9.01, 16.04)	2593.73 (2258.83, 3056.82)		186.50 (162.42, 219.80)	10.20 (9.00, 11.40)	
Indonesia	1.15 (0.00, 2.00)	0.62 (0.00, 1.08)	419.40 (339.28, 523.82)		161.64 (130.76, 201.89)	17.00 (13.50, −20.50)	
Italy	107.53 (104.67, 110.67)	189.31 (184.29, 194.85)	32.63 (29.17, 37.80)		54.10 (48.36, 62.67)	−4.40 (−5.90, −2.80)	
Japan	3.46 (3.23, 3.89)	2.75 (2.56, 3.09)	9.06 (7.12, 11.54)		7.09 (5.57, 9.03)	3.30 (2.40, −4.20)	
Mexico	113.96 (111.72, 116.20)	133.30 (130.68, 135.92)	264.70 (256.17, 276.13)		211.86 (205.03, 221.01)	1.50 (1.10, −1.90)	
Republic of Korea	2.83 (2.35, 3.16)	6.37 (5.30, 7.13)	8.89 (6.63, 12.55)		16.64 (12.43, 23.51)	3.40 (1.80, −5.10)	
Russian Federation	187.52 (183.61, 194.63)	124.17 (121.58, 128.87)	1071.67 (1030.50, 1124.36)		730.43 (702.37, 766.35)	6.50 (5.30, −7.60)	
Saudi Arabia	4.32 (2.31, 8.23)	26.95 (14.38, 51.31)	35.56 (14.00, 96.90)		99.51 (39.18, 271.19)	4.60 (4.50, 4.80)	
South Africa	207.50 (154.30, 303.88)	563.42 (418.94, 825.09)	7682.98 (6492.02, 9535.64)		13821.18 (11678.72, 17153.99)	11.70 (10.30, 13.10)	
Turkey	0.50 (0.00, 0.75)	0.83 (0.00, 1.25)	11.78 (9.54, 14.09)		14.48 (11.73, 17.32)	10.10 (9.60, 10.60)	
UK	24.44 (23.56, 25.82)	42.52 (40.99, 44.93)	20.30 (15.28, 27.10)		30.20 (22.73, 40.32)	−1.60 (−3.10, −0.10)	
USA	1488.92 (1442.34, 1549.37)	587.10 (568.73, 610.94)	415.33 (346.51, 508.43)		126.63 (105.65, 155.02)	−5.60 (−6.50, −4.60)	

AAPC – annual percent change, ASDR – age-standardised DALY rate, DALY – disability-adjusted life years, G20 – Group of Twenty, UI – uncertainty interval

In 2019, the G20 reported an incidence of 0.80 million cases of AIDS, with an ASIR of 16.48 (95% UI = 14.19, 18.87). China’s ASIR was 2.24 (95% UI = 1.08, 3.62), with the AAPC of 3.20 (95% UI = 1.80, 4.70) (Table S1 in the **Online Supplementary Document**, **Figure 2**, panel A, **Figure 3**). The mortality was 0.28 million in G20, and the ASMR was 5.77 (95% UI = 5.36, 6.45) (Table S2 in the **Online Supplementary Document**, **Figure 2**, panel A, **Figure 3**). During the period, China had a lower ASDR compared to G20 countries, but the AAPC of ASDR in China was 1.76 times higher than that of the G20 countries (Table S2 in the **Online Supplementary Document****,** **Figure 2**, panel B, **Figure 3**). Although the worldwide ASIR was higher than G20 and countries, the AAPC was significantly lower with a value of −1.40 (95% UI = −1.60, −1.20) (Table S3 in the **Online Supplementary Document**, **Figure 3**). The same trend was observed for worldwide ASDR, DALY and their AAPC (Tables S4-5 in the **Online Supplementary Document**, **Figure 3**).

The MIR in G20 was 0.35 (95% UI = 0.30, 0.42) in 2019, which increased of 45.83% compared with 1990. The AAPC of MIR was 1.10 (95% UI = 0.30, 0.42) (**Table 2**, **Figure 2**, panel A, **Figure 3**). Among the G20 countries, the highest AAPC of MIR was found in Indonesia, and the lowest was found in Australia with a value of −7.20 (95% UI = −9.20, 5.20) (**Table 2**, **Figure 3**). However, China’s MIR in 2019 ranked first among G20, although it was at a moderate level in 1990. The AAPC of China’s MIR ranked 4th with a value of 4.60 (95% UI = 3.10, 6.00) (**Table 2**, **Figure 2**, panel B, **Figure 3**). In terms of age, within the G20, only the 45 − 54 and 75 − 79 age groups had MIR values greater than 1 (Figure S1 in the **Online Supplementary Document**). However, in China, all age groups over 35 had MIR values greater than 1, with some age groups even exceeding 2 (**Table 2**, **Figure 2**, S1 in the **Online Supplementary Document**).

**Table 2:** The MIR and temporal trend from 1990 to 2019 in China and G20

Characteristics	MIR in 1990	MIR in 2019	AAPC
			**No. (95% UI)**
**China**			
**Total**	0.28 (0.11, 0.71)	1.00 (0.61, 2.31)	4.60 (3.10, 6.00)
**Male**	0.28 (0.10, 0.74)	0.96 (0.58, 2.14)	4.50 (3.00, 6.00)
**Female**	0.28 (0.11, 0.72)	1.13 (0.71, 2.42)	4.80 (3.10, 6.50)
**Age**			
15 to 19	0.05 (0.02, 0.11)	0.08 (0.04, 0.29)	1.40 (0.20, 2.60)
20 to 24	0.08 (0.03, 0.21)	0.10 (0.06, 0.28)	0.60 (−0.30, 1.50)
25 to 29	0.18 (0.05, 0.58)	0.37 (0.19, 1.93)	3.00 (1.80, 4.20)
30 to 34	0.26 (0.08, 0.87)	0.88 (0.52, 2.40)	4.60 (3.60, 5.70)
35 to 39	0.30 (0.05, 1.30)	1.62 (0.71, 6.83)	6.30 (5.20, 7.30)
40 to 44	0.39 (0.07, 1.78)	2.26 (1.35, 5.34)	6.10 (3.80, 8.50)
45 to 49	0.53 (0.08, 2.23)	2.94 (1.56, 7.94)	5.70 (2.30, 9.30)
50 to 54	0.43 (0.12, 1.57)	2.22 (1.31, 4.90)	5.40 (2.90, 7.90)
55 to 59	0.41 (0.12, 1.22)	1.51 (0.93, 3.18)	4.50 (2.70, 6.40)
60 to 64	0.38 (0.11, 1.30)	1.02 (0.65, 2.06)	3.10 (1.30, 5.00)
65 to 69	0.54 (0.19, 1.43)	1.22 (0.76, 2.79)	2.80 (0.70, 5.10)
70 to 74	0.38 (0.11, 0.78)	1.95 (1.19, 4.24)	6.20 (3.70, 8.70)
75 to 79	0.12 (0.00, 0.86)	1.48 (0.88, 3.35)	10.30 (6.70, 14.10)
**G20**			
**Total**	0.24 (0.21, 0.29)	0.35 (0.30, 0.42)	1.10 (0.40, 1.80)
**Male**	0.30 (0.25, 0.37)	0.36 (0.30, 0.43)	0.40 (−0.70, 1.50)
**Female**	0.13 (0.11, 0.16)	0.34 (0.28, 0.44)	3.30 (2.70, 4.00)
**Age**			
15 to 19	0.03 (0.02, 0.04)	0.11 (0.08, 0.15)	4.70 (4.10, 5.20)
20 to 24	0.05 (0.04, 0.07)	0.10 (0.07, 0.14)	2.30 (1.40, 3.20)
25 to 29	0.14 (0.12, 0.17)	0.13 (0.08, 0.18)	−0.60 (−2.00, 0.90)
30 to 34	0.26 (0.22, 0.32)	0.23 (0.16, 0.30)	−0.60 (−1.40, 0.20)
35 to 39	0.37 (0.31, 0.45)	0.39 (0.26, 0.61)	0.10 (−1.10, 1.40)
40 to 44	0.50 (0.43, 0.61)	0.72 (0.55, 1.00)	1.20 (−0.10, 2.50)
45 to 49	0.57 (0.48, 0.68)	1.03 (0.71, 1.58)	2.00 (1.00, 3.10)
50 to 54	0.45 (0.39, 0.51)	1.06 (0.80, 1.59)	2.90 (1.60, 4.20)
55 to 59	0.50 (0.44, 0.58)	0.68 (0.47, 1.08)	0.90 (−0.40, 2.20)
60 to 64	0.54 (0.46, 0.65)	0.59 (0.42, 0.85)	0.30 (−0.40, 1.00)
65 to 69	0.79 (0.66, 0.99)	0.60 (0.46, 0.82)	−1.10 (−1.60, 0.50)
70 to 74	0.23 (0.17, 0.30)	0.74 (0.56, 0.97)	4.10 (3.20, 4.90)
75 to 79	0.10 (0.05, 0.45)	1.02 (0.77, 1.43)	7.30 (6.30, 8.40)
**Regions**			
Argentina	0.11 (0.08, 0.17)	0.15 (0.10, 0.33)	1.30 (0.30, 2.20)
Australia	0.33 (0.28, 0.40)	0.05 (0.03, 0.09)	−7.20 (−9.20, 5.20)
Brazil	0.30 (0.26, 0.36)	0.24 (0.20, 0.31)	−0.80 (−1.80, 0.10)
Canada	0.29 (0.20, 0.53)	0.08 (0.05, 0.16)	−4.50 (−7.30, −1.60)
China	0.28 (0.11, 0.71)	1.00 (0.61, 2.31)	4.60 (3.10, 6.00)
European Union	0.38 (0.29, 0.53)	0.14 (0.12, 0.19)	−4.10 (−5.10, −3.00)
France	0.84 (0.76, 0.95)	0.18 (0.12, 0.34)	−5.80 (−7.40, −4.10)
Germany	0.56 (0.47, 0.72)	0.18 (0.13, 0.32)	−3.50 (−6.60, −0.40)
India	0.04 (0.02, 0.19)	0.63 (0.41, 1.29)	10.10 (8.00, 12.30)
Indonesia	0.00 (0.00, 0.02)	0.40 (0.27, 0.57)	16.30 (9.40, 23.70)
Italy	0.30 (0.00, 2.47)	0.16 (0.11, 0.30)	−7.00 (−9.70, −4.30)
Japan	0.11 (0.07, 0.27)	0.06 (0.04, 0.12)	−2.10 (−4.40, 0.20)
Mexico	0.12 (0.09, 0.18)	0.30 (0.24, 0.39)	3.20 (2.00, 4.50)
Republic of Korea	0.15 (0.00, 1.25)	0.11 (0.06, 0.48)	−6.60 (−9.70, −3.40)
Russian Federation	0.50 (0.41, 0.64)	0.16 (0.13, 0.20)	−3.70 (−6.30, −1.10)
Saudi Arabia	0.30 (0.11, 1.22)	0.66 (0.00, 4.79)	2.60 (2.30, 2.90)
South Africa	0.05 (0.03, 0.07)	0.39 (0.29, 0.54)	7.80 (7.10, 8.50)
Turkey	0.20 (0.00, 2.15)	0.51 (0.34, 0.77)	2.30 (1.20, 3.30)
UK	0.22 (0.15, 0.36)	0.04 (0.03, 0.08)	−6.00 (−8.50, −3.40)
USA	0.47 (0.34, 0.76)	0.11 (0.07, 0.25)	−5.60 (−8.20, −2.90)

AAPC – annual percent change, G20 – Group of Twenty, MIR – mortality-incidence ratio, UI – uncertainty interval

### Gender and age disparities in DALY and MIR of AIDS

The incidence, mortality, DALY, as well as their ASR were all higher in male compared to female in 1990 and 2019 (**Table 1**, Tables S1–5 in the **Online Supplementary Document**, **Figure 1**). In addition, the gender gap in China’s DALY widened during the period. The ratio of DALY in male to DALY in female increased from 1.76 in 1990 to 2.85 in 2019. Conversely, the ratio decreased from 3.79 to 1.20 in G20 (**Table 1**, **Figure 1**). The AAPC of ASDR in China was higher both in male and female compared to the G20. Within China, the AAPC of ASDR in male was higher than female. In contrast, the AAPC was more than two times higher in female in G20 compared to male (**Table 1**, **Figure 1**). The MIR in China was higher both in male and female compared to G20, with a higher AAPC at the same time (**Table 2**, **Figure 2**). Additionally, in terms of gender, both China and the G20 showed an upward trend in MIR, with China experiencing a greater increase both in male and female (**Table 2**, **Figure 2**).

In terms of the age distribution of DALYs, there were similarities between China and G20 countries. In 1990, the majority of G20 countries had the highest proportion of DALYs in the 25–49 age group. However, by 2019, there was an increase in the proportion of DALY in the 50–69 age group, accompanied by a decrease in the 25–49 age group, particularly notable in Japan (**Figure 1**, **Figure 2**). In 2019, China and the G20 had the highest ASDR in the 30–49 age group. Additionally, the lowest ASDR in China in 2019 was found in the 15–19 age group, followed by the 20–24 age group, and was found in the 70–74 age group, followed by the 75–79 age group in the G20. It is worth noting that over the past thirty years, China has experienced a consistent upward trend in ASDR, whereas the G20 initially witnessed an increase, followed by a subsequent decrease (**Figure 2**).

### Joinpoint regression analysis of AIDS ASR trend

**Figure 4:**
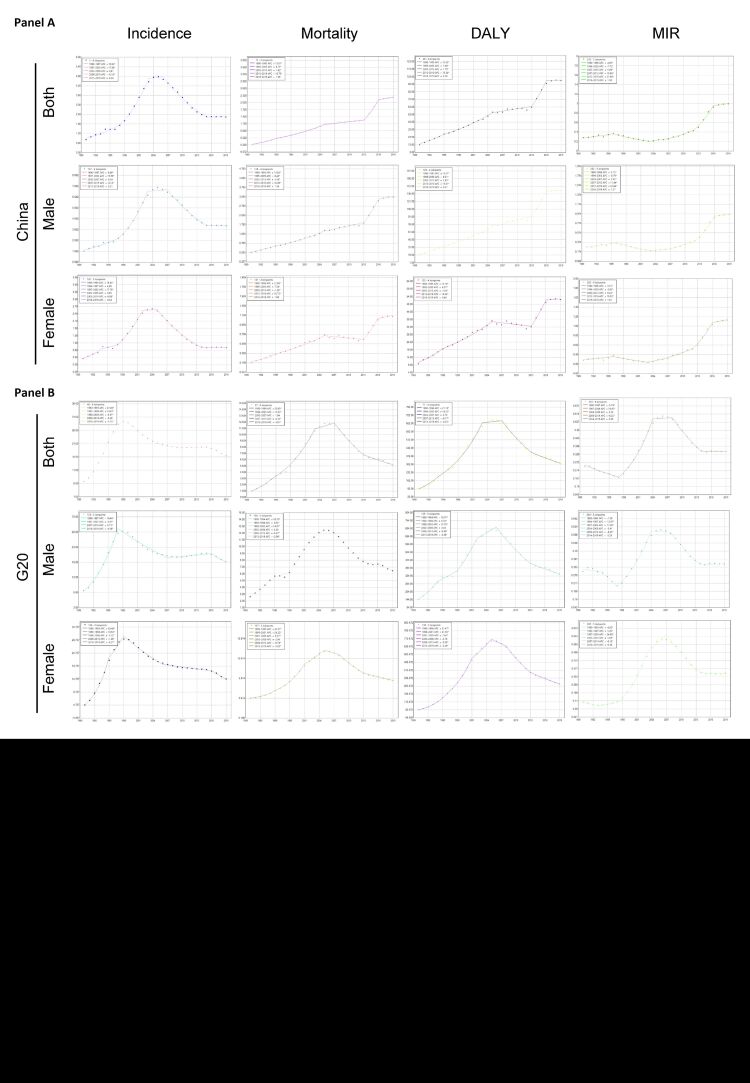
Joinpoint regression analysis of age-standardised incidence rate (ASIR), age-standardised mortality rate (ASMR), age-standardised DALY rate (ASDR) and age-standardised rate (ASR) of mortality-incidence ratio (MIR) for AIDS in China (**Panel A**) and the Group of Twenty (G20) countries (**Panel B**) among males, females, and both sexes from 1990 to 2019.

In China, significant changes in ASDR occurred in 1995, 2005, 2013, and 2016, while in the G20 countries, significant changes were observed in 1994, 2003, 2007, and 2013 (**Figure 4**). The time period with the most significant increase of ASDR in China, for both gender and overall, was 2013–2016, followed by 1990 − 1995 (**Figure 4**). In the G20, the time period was 1990–1994, which was earlier than the corresponding period in China. The most notable period of decrease in ASDR in China was 2016–2019, while the G20 has been experiencing a continuous decline since 2007, with the most significant decrease occurring 2007–2013 (**Figure 4**).

Over the past thirty years, both China and the G20 exhibited a trend of initially increasing and then decreasing in ASIR. The fastest increase in ASIR for China occurred from 1997 to 2003, while the most rapid decline occurred from 2006 to 2015. On the other hand, the G20 countries experienced a growth in ASIR until 1998, after which a downward trend began to emerge (**Figure 4**). The time periods of significant change in ASMR were similar to those of ASDR (**Figure 4**).

Additionally, significant changes in MIR of AIDS in China were in 1996, 2003, 2007, 2012 and 2016, while in the G20, they occurred in 1997, 2004, 2008, and 2014 (**Figure 4**). Similar to ASDR, the time period with the significant increase in China was 2012 − 2016, while the G20 has been experiencing a continuous decline since 2008, with the most significant decrease occurring 2008–2014 (**Figure 4**).

### Association between AAPC and SDI, HAQ

**Figure 5:**
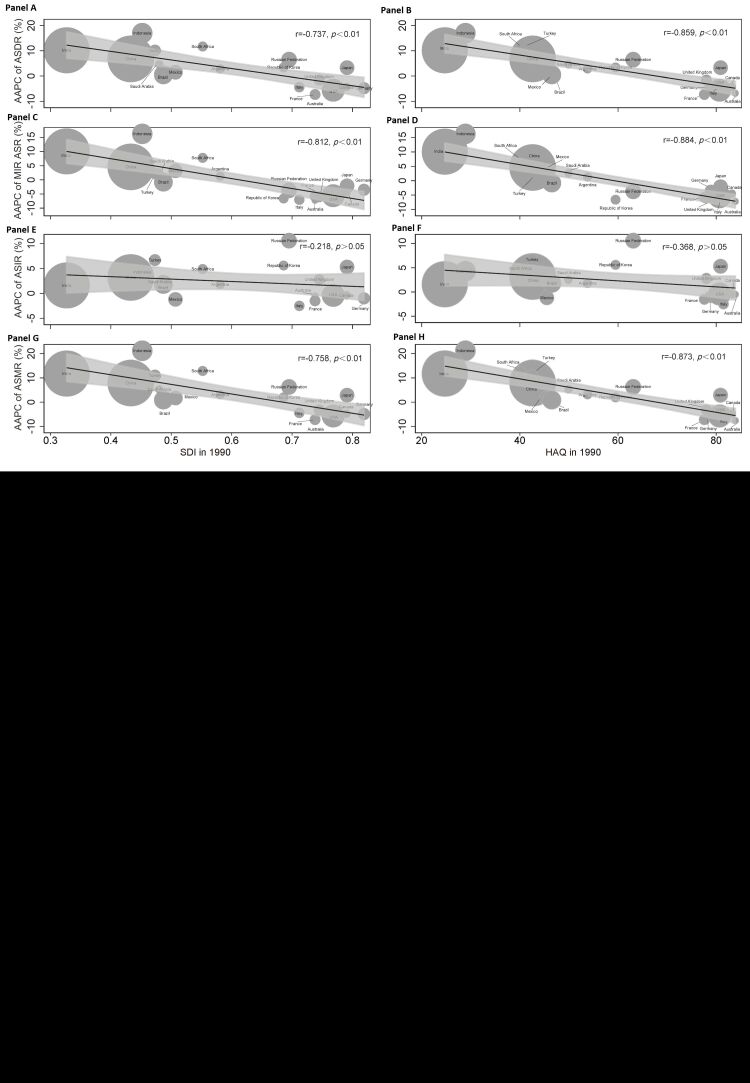
Correlation between average annual percentage changes (AAPC) for AIDS and social development index (SDI) or health care access and quality (HAQ) in the Group of Twenty (G20) countries. **Panel A.** The AAPC of age-standardised disability-adjusted life years (DALY) rate (ASDR) was negatively correlated with SDI. **Panel B.** The AAPC of ASDR was negatively correlated with HAQ. **Panels C** and **D.** The AAPC of mortality-incidence ratio (MIR) age-standardised rate (ASR) was also negatively correlated with SDI and HAQ. **Panels E** and (**F**). The AAPC of age-standardised incidence rate (ASIR) did not show any significant corelation with SDI and HAQ. **Panels G** and (**H**). The AAPC of age-standardised mortality rate (ASMR) was negatively corelated with SDI and HAQ.

The analysis revealed a negative relationship between the AAPC of ASDR and SDI (r = −0.737, *P* < 0.01) (**Figure 5**, panel A). Similarly, a negative association was observed between AAPC of ASDR and HAQ (r = −0.859, *P* < 0.01) (**Figure 5**, panel B). Interestingly, Japan exhibited a higher SDI and HAQ scores compared to most of the G20 countries, however, its AAPC of ASDR was higher than that of similar countries (**Figure 5**, panels A and B). Moreover, France and Brazil obtained a lower AAPC of ASDR compared to countries with similar SDI or HAQ scores (**Figure 5**, panels A and B).

Similar to the trend observed in the AAPC of ASDR, the AAPC of MIR ASR showed a negative correlation with SDI and HAQ (**Figure 5**, panels C and D). Notably, Indonesia had a higher AAPC of MIR ASR compared to the average level, while the Republic of Korea had a lower AAPC of MIR ASR than the average level (**Figure 5**, panels C and D). Although the AAPC of ASIR did not show any correlation with SDI or HAQ, there was a significant negative correlation observed in the AAPC of ASDR (**Figure 5**, panels E–H).

### Prediction of cases and ASR from 2020 to 2040

**Figure 6:**
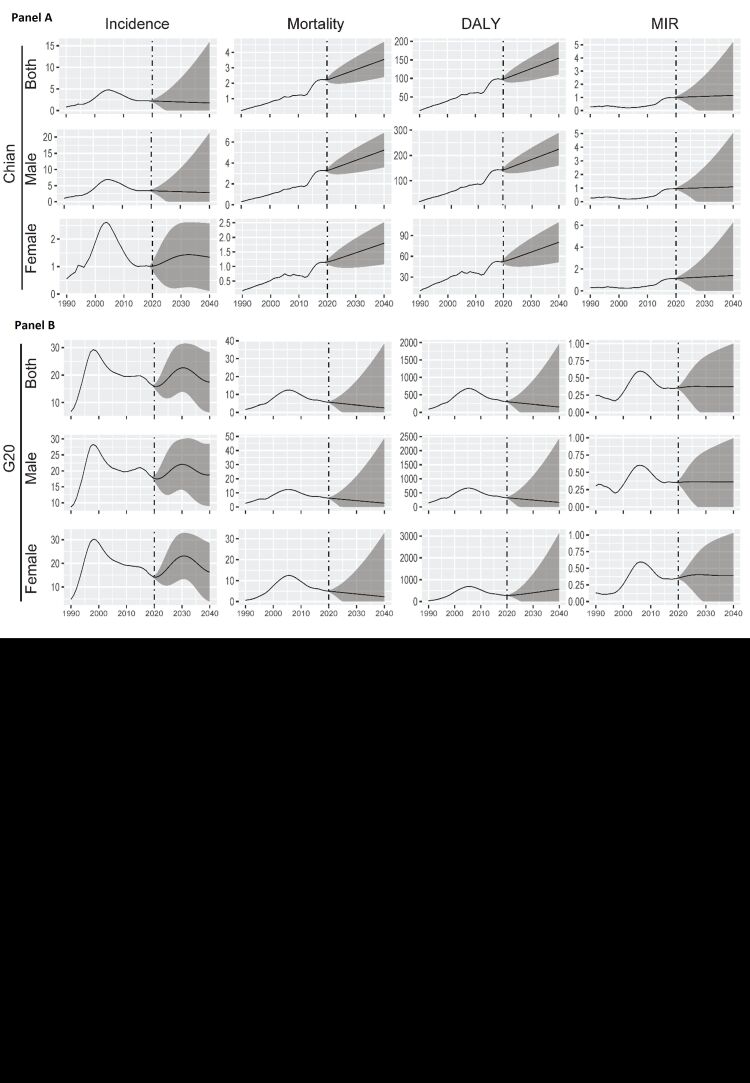
Prediction of age-standardised incidence rate (ASIR), age-standardised mortality rate (ASMR), age-standardised disability-adjusted life years (DALY) rate (ASDR), and age-standardised rate (ASR) of mortality-incidence ratio (MIR) for AIDS among males, females, and both sexes in China (**Panel A**) and the Group of Twenty (G20) countries (**Panel B**).

The DALY of G20 in 2040 was 9424.24 (95% UI = 0.00, 87759.80). The ASR was 153.77 (95% UI = 0.00, 1971.38), representing a significant decrease compared to 2019 (**Table 3**, **Figure 6**). Moreover, the ASDR exhibited a worldwide decrease during the predicted period (Table S11 in the **Online Supplementary Document**). Over the next 20 years, the ASDR was expected to either remain stable or decrease in higher SDI countries, while an increase was predicted in lower SDI countries (**Table 3**). In China, the ASDR in 2040 was 154.74 (95% UI = 110.85–198.64), which increased of 57.66% compared to 2019 (**Table 3**, **Figure 6**, panel A). In term of gender, the ASDR for male in G20 in 2040 significantly decrease from 335.51 to 163.70, whereas for females, it increased from 283.11 in 2019 to 567.81 in 2040. In China, the ASDR increased both in male and female, deviating from the overall trend observed in G20 (**Table 3**, Figure 6, panel A). In term of age, the ASDR increased in all age group except for the 65–69 age group. However, it decreased in the age group older than 30 (excluding the 70–74 age group) in G20 countries (**Table 3**).

**Table 3:** The prediction of DALY in next 20 y in China and G20

Characteristics	2030		2040	
	**DALY cases**	**ASDR per 100 000**	**DALY cases**	**ASDR per 100 000**
	**No. ×10^3^ (95% UI)**	**No. (95% UI)**	**No. ×10^3^ (95% UI)**	**No. (95% UI)**
**China**				
**Total**	1804.07 (1366.77, 2241.38)	126.15 (95.07, 157.24)	2220.07 (1602.01, 2838.14)	154.74 (110.85, 198.64)
**Male**	1332.20 (998.32, 1666.08)	183.01 (137.69, 228.32)	1641.68 (1166.30, 2117.05)	224.83 (160.51, 289.15)
**Female**	464.58 (326.68, 602.47)	66.00 (45.78, 86.22)	567.84 (370.04, 765.63)	80.29 (51.36, 109.23)
**Age**				
15 to 19	17.14 (7.32, 26.96)	29.84 (20.62, 39.05)	17.55 (0.29, 34.80)	39.26 (16.40, 62.13)
20 to 24	40.48 (12.39, 68.58)	67.94 (53.79, 82.08)	40.36 (0.00, 87.93)	82.83 (61.77, 103.89)
25 to 29	144.72 (91.41, 198.02)	140.83 (119.67, 161.99)	176.20 (100.38, 252.02)	171.56 (142.00, 201.11)
30 to 34	270.98 (182.79, 359.17)	201.55 (157.20, 245.91)	333.60 (204.64, 462.57)	247.02 (184.80, 309.25)
35 to 39	228.74 (167.31, 290.17)	224.01 (176.87, 271.15)	281.64 (195.36, 367.92)	275.08 (208.92, 341.24)
40 to 44	220.80 (153.09, 288.51)	211.37 (154.91, 267.83)	272.50 (179.89, 365.11)	259.24 (181.46, 337.02)
45 to 49	250.94 (184.68, 317.19)	204.93 (155.86, 254.01)	311.17 (219.07, 403.27)	252.13 (183.20, 321.06)
50 to 54	150.48 (25.56, 275.41)	162.00 (106.26, 217.73)	150.37 (0.00, 342.38)	199.54 (120.59, 278.50)
55 to 59	167.30 (96.67, 237.93)	111.92 (79.72, 144.13)	245.67 (72.19, 419.16)	137.05 (91.87, 182.23)
60 to 64	82.37 (50.66, 114.09)	99.82 (64.02, 135.63)	103.30 (60.38, 146.22)	122.10 (71.81, 172.39)
65 to 69	65.27 (0.00, 166.60)	82.68 (15.09, 150.26)	65.02 (0.00, 328.51)	82.63 (0.00, 183.98)
70 to 74	82.79 (27.93, 137.66)	102.87 (69.25, 136.50)	124.07 (0.00, 268.48)	126.97 (79.17, 174.77)
75 to 79	21.65 (13.99, 29.31)	69.22 (43.98, 94.47)	26.62 (15.76, 37.47)	83.13 (47.65, 118.60)
**G20**				
**Total**	12104.19 (0.00, 41902.62)	227.92 (0.00, 919.34)	9424.24 (0.00, 87759.80)	153.77 (0.00, 1971.38)
**Male**	6556.22 (0.00, 24946.35)	245.52 (0.00, 1111.84)	5049.59 (0.00, 53408.68)	163.70 (0.00, 2441.84)
**Female**	9656.69 (0.00, 28529.14)	401.25 (0.00, 1274.15)	13427.78 (0.00, 68685.18)	567.81 (0.00, 3146.14)
**Age**				
15 to 19	495.54 (0.00, 1222.26)	152.85 (0.00, 374.55)	546.99 (0.00, 2481.99)	177.04 (0.00, 772.27)
20 to 24	703.66 (0.00, 2887.75)	314.01 (104.54, 523.47)	734.10 (0.00, 6947.90)	311.58 (74.50, 548.67)
25 to 29	629.13 (0.00, 4582.73)	421.13 (0.00, 1982.55)	0.00 (0.00, 10204.02)	477.99 (0.00, 4869.02)
30 to 34	1661.38 (0.00, 7958.28)	310.86 (0.00, 2148.88)	974.26 (0.00, 17447.92)	30.78 (0.00, 4837.37)
35 to 39	2156.26 (0.00, 8872.53)	491.88 (0.00, 2593.89)	1784.50 (0.00, 19404.81)	277.57 (0.00, 5792.32)
40 to 44	1948.53 (0.00, 7823.34)	487.58 (0.00, 2247.46)	1847.64 (0.00, 17742.80)	348.55 (0.00, 4977.55)
45 to 49	1821.16 (0.00, 5329.00)	443.69 (0.00, 1823.29)	1942.44 (0.00, 10981.20)	393.89 (0.00, 4020.14)
50 to 54	1093.55 (0.00, 2944.17)	299.78 (0.00, 1037.33)	1032.87 (0.00, 5893.89)	240.85 (0.00, 2174.25)
55 to 59	740.74 (228.64, 1252.83)	166.88 (0.00, 695.86)	750.56 (0.00, 1665.79)	93.77 (0.00, 1515.09)
60 to 64	517.24 (376.54, 657.93)	167.11 (0.00, 485.91)	634.06 (430.88, 837.23)	166.92 (0.00, 1032.13)
65 to 69	313.56 (224.85, 402.26)	107.20 (0.00, 245.83)	385.08 (261.15, 509.00)	96.22 (0.00, 450.82)
70 to 74	154.36 (96.68, 212.05)	74.73 (0.00, 182.66)	190.65 (108.54, 272.75)	77.97 (0.00, 361.06)
75 to 79	47.04 (13.04, 81.04)	40.96 (0.00, 126.45)	47.14 (0.00, 104.51)	36.44 (0.00, 261.30)
**Regions**				
Argentina	99.45 (0.00, 384.54)	223.11 (103.24, 342.99)	96.70 (0.00, 825.96)	223.39 (102.09, 344.69)
Australia	4.29 (0.00, 21.53)	17.48 (0.00, 113.46)	4.29 (0.00, 28.11)	17.48 (0.00, 150.10)
Brazil	815.47 (557.57, 1073.37)	350.26 (0.00, 839.15)	814.75 (536.46, 1093.05)	348.78 (0.00, 1141.72)
Canada	17.72 (0.00, 87.67)	48.39 (0.00, 288.37)	17.72 (0.00, 115.60)	48.39 (0.00, 384.24)
China	1804.07 (1366.77, 2241.38)	126.15 (95.07, 157.24)	2220.07 (1602.01, 2838.14)	154.74 (110.85, 198.64)
European Union	213.10 (0.00, 1046.39)	41.39 (0.00, 213.49)	213.10 (0.00, 1383.00)	41.39 (0.00, 283.01)
France	25.70 (0.00, 339.14)	38.62 (0.00, 575.79)	25.69 (0.00, 490.43)	38.61 (0.00, 836.12)
Germany	22.97 (0.00, 131.26)	27.03 (0.00, 159.97)	22.97 (0.00, 175.81)	27.03 (0.00, 214.88)
India	8187.38 (0.00, 21675.56)	604.96 (0.00, 1785.34)	16323.17 (0.00, 60365.24)	1240.02 (0.00, 5138.17)
Indonesia	583.69 (523.86, 643.52)	225.01 (198.34, 251.67)	729.45 (647.29, 811.62)	281.36 (244.69, 318.03)
Italy	32.27 (0.00, 286.65)	53.58 (0.00, 503.67)	32.27 (0.00, 387.66)	53.58 (0.00, 682.28)
Japan	9.06 (4.09, 14.03)	7.09 (3.23, 10.95)	9.06 (2.19, 15.93)	7.09 (1.75, 12.43)
Mexico	231.30 (0.00, 472.16)	210.39 (85.55, 335.24)	200.93 (0.00, 786.21)	215.76 (87.46, 344.06)
Republic of Korea	11.19 (7.84, 14.53)	20.54 (13.47, 27.60)	13.28 (8.65, 17.90)	24.08 (14.32, 33.84)
Russian Federation	1407.03 (1082.10, 1731.96)	960.39 (739.19, 1181.59)	1711.91 (1262.95, 2160.86)	1169.44 (863.82, 1475.07)
Saudi Arabia	33.91 (24.54, 43.27)	65.12 (17.59, 112.65)	32.64 (10.57, 54.71)	30.49 (0.00, 166.65)
South Africa	16517.67 (0.00, 43975.06)	27837.21 (0.00, 79785.56)	24912.49 (0.00, 99369.99)	41213.35 (0.00, 181954.80)
Turkey	16.14 (12.29, 19.98)	19.72 (14.55, 24.89)	20.03 (14.63, 25.42)	24.43 (17.17, 31.69)
UK	20.38 (0.00, 60.30)	30.16 (0.00, 99.42)	20.38 (0.00, 77.83)	30.16 (0.00, 130.08)
USA	277.67 (0.00, 2270.94)	75.69 (0.00, 815.74)	277.67 (0.00, 3120.37)	75.69 (0.00, 1131.51)

ASDR – age-standardised DALY rate, DALY – disability-adjusted life years, G20 – Group of Twenty, UI – uncertainty interval

The ASIR decreased in China over the next 20 years, while it initially increased and then decreased in G20 countries. However, at the global level, the ASIR showed an increase during the predicted period (Tables S7–S9 in the **Online Supplementary Document**, **Figure 6**). In contrast, the ASMR significantly increased in China from 2020 to 2040, while it decreased in G20 countries and remained stable worldwide (Table S8–S10 in the **Online Supplementary Document**, **Figure 6**). The ASR of MIR exhibited a mild increase or remains stable in China, G20 countries, and globally from 2020 to 2040 (**Table 4**, Table S12 in the **Online Supplementary Document**, **Figure 6**).

**Table 4:** The prediction of MIR in next 20 y in China and G20

Characteristics	2030	2040
	**MIR**	**MIR**
**China**		
**Total**	1.08 (0.00, 2.67)	1.16 (0.00, 5.22)
**Male**	1.03 (0.00, 2.58)	1.09 (0.00, 5.05)
**Female**	1.28 (0.00, 3.18)	1.41 (0.00, 6.27)
**Age**		
15 to 19	0.10 (0.04, 0.16)	0.12 (0.00, 0.25)
20 to 24	0.10 (0.06, 0.13)	0.10 (0.05, 0.14)
25 to 29	0.36 (0.18, 0.54)	0.36 (0.11, 0.62)
30 to 34	0.90 (0.00, 2.37)	0.92 (0.00, 4.70)
35 to 39	1.82 (0.00, 4.52)	2.00 (0.00, 8.90)
40 to 44	2.38 (0.00, 5.72)	2.49 (0.00, 11.02)
45 to 49	2.83 (0.00, 7.47)	2.72 (0.00, 14.61)
50 to 54	2.00 (0.00, 5.76)	1.80 (0.00, 11.42)
55 to 59	1.44 (0.00, 4.27)	1.38 (0.00, 8.61)
60 to 64	0.88 (0.00, 1.91)	0.87 (0.00, 2.48)
65 to 69	1.01 (0.00, 3.91)	0.82 (0.00, 8.23)
70 to 74	2.14 (0.00, 6.21)	2.31 (0.00, 12.89)
75 to 79	0.00 (0.00, 3.79)	0.00 (0.00, 9.32)
**G20**		
**Total**	0.37 (0.00, 0.82)	0.37 (0.00, 1.00)
**Male**	0.36 (0.00, 0.81)	0.36 (0.00, 0.99)
**Female**	0.40 (0.00, 0.85)	0.39 (0.00, 1.03)
**Age**		
15 to 19	0.16 (0.09, 0.24)	0.21 (0.02, 0.40)
20 to 24	0.11 (0.00, 0.24)	0.11 (0.00, 0.30)
25 to 29	0.18 (0.00, 0.37)	0.17 (0.00, 0.36)
30 to 34	0.41 (0.16, 0.67)	0.32 (0.04, 0.61)
35 to 39	0.53 (0.00, 1.20)	0.46 (0.00, 1.26)
40 to 44	0.79 (0.00, 1.70)	0.76 (0.00, 1.84)
45 to 49	1.04 (0.00, 2.14)	1.04 (0.00, 2.62)
50 to 54	0.97 (0.10, 1.85)	0.98 (0.00, 2.22)
55 to 59	0.71 (0.06, 1.36)	0.71 (0.00, 1.67)
60 to 64	0.53 (0.35, 0.71)	0.55 (0.35, 0.74)
65 to 69	0.59 (0.45, 0.74)	0.61 (0.43, 0.80)
70 to 74	0.88 (0.45, 1.31)	0.89 (0.19, 1.59)
75 to 79	0.62 (0.00, 1.37)	0.67 (0.00, 1.59)
**Regions**		
Argentina	0.21 (0.10, 0.32)	0.20 (0.08, 0.31)
Australia	0.05 (0.00, 0.66)	0.05 (0.00, 0.94)
Brazil	0.24 (0.00, 0.49)	0.25 (0.00, 0.56)
Canada	0.08 (0.00, 0.83)	0.08 (0.00, 1.14)
China	1.08 (0.00, 2.67)	1.16 (0.00, 5.22)
European Union	0.15 (0.00, 1.03)	0.15 (0.00, 1.39)
France	0.19 (0.00, 2.60)	0.19 (0.00, 3.57)
Germany	0.18 (0.00, 0.90)	0.18 (0.00, 1.21)
India	1.17 (0.00, 2.52)	0.93 (0.00, 2.58)
Indonesia	0.55 (0.30, 0.80)	0.69 (0.33, 1.05)
Italy	0.16 (0.00, 3.32)	0.16 (0.00, 4.59)
Japan	0.06 (0.00, 0.17)	0.06 (0.00, 0.22)
Mexico	0.48 (0.20, 0.76)	0.40 (0.08, 0.71)
Republic of Korea	0.10 (0.03, 0.18)	0.10 (0.02, 0.19)
Russian Federation	0.15 (0.00, 1.27)	0.15 (0.00, 1.72)
Saudi Arabia	0.63 (0.26, 1.01)	0.63 (0.02, 1.24)
South Africa	0.63 (0.00, 1.38)	0.85 (0.00, 2.89)
Turkey	0.52 (0.24, 0.79)	0.52 (0.13, 0.90)
UK	0.04 (0.00, 0.45)	0.04 (0.00, 0.62)
USA	0.11 (0.00, 1.27)	0.11 (0.00, 1.71)

G20 – Group of Twenty, MIR – mortality-incidence ratio

## DISCUSSION

AIDS not only poses significant challenges to global human health, but also imposes a heavy burden on the economy. In our study, we first examined the DALY trend of AIDS in China and G20, compared and predicted the trend for the next 20 years. By studying the burden of AIDS, we have assessed the extent of impact on populations across different countries, genders, and age groups, and provided scientific evidence and recommendations for the development of AIDS prevention and control measures.

In term of countries, over the past 30 years, the DALY trends of AIDS varied among G20 countries. In 1990, the United States had the highest DALY and ASDR of AIDS among the G20. However, significant progress has been made through comprehensive HIV prevention measures, including promoting safe sexual practices, providing condoms and needles, and strengthening monitoring and treatment of HIV-infected individuals such as establishing the STARHS surveillance system., improving the AIDS case reporting system, and establishing the HANC, as well as enhancing HIV knowledge dissemination and educational campaigns such as incorporating relevant courses into the school education system, creating promotional materials, and utilising social media for knowledge dissemination [13–17]. Furthermore, enhancing HIV knowledge dissemination and educational campaigns, such as integrating related courses into the school curriculum, played a key role in controlling the AIDS epidemic and reducing the ASDR [14,16].

Contrarily, there has been a significant increase in the ASDR of AIDS in Southeast Asia and Africa, with particularly high and persistent in South Africa. The escalating AIDS epidemic in these two regions can be attributed to various factors, including a lack of relevant knowledge, limited access to antiretroviral therapy, insufficient social protection, and a lack of social support and services [18–21]. In addition, Indonesia had experienced a notable increase in DALY and ASDR of AIDS, which may be attributed to a lack of care to high-risk populations and limited funding for AIDS prevention and control [22].

China has made significant efforts in the prevention and control of AIDS, including policy and system improvements, expansion of prevention and treatment teams, and increased public awareness and education [23]. Although certain achievements have been made in China, the DALY and ASDR are still increasing, especially during the period of 1990–1995 and 2013–2016. Before 1994, China lacked specific prevention and control measures for HIV/AIDS, leading to the initial significant increase in the disease burden. Since 1995, China has established HIV sentinel surveillance system, which had expanded to 393 monitoring sites by 2006, covering key populations[24,25]. In 2004, the issuance of the ‘Notice of the State Council on Strengthening HIV/AIDS Prevention and Control’ marked a significant turning point in China's fight against AIDS. The ‘Four Frees and One Care’ policy introduced by this notice effectively changed the passive situation of AIDS prevention and alleviated the disease burden [24,25]. Additionally, in 2010, the ‘Five Expansions and Six Enhancements’ measures proposed in 2010 further slowed the growth of ASMR and ASDR in China [26]. However, the significant increase in ASMR and ASDR between 2013 and 2016 may be associated with the improper use and incomplete coverage of antiretroviral therapy among the population [27].

In term of gender, our study indicated the DALY were generally higher in males, with the disparity between men and women increasing in China and decreasing in G20 countries. Studies indicated that HIV infection itself did not have gender differences, and the gender disparities in burden were mainly associated with changes in the regional patterns of AIDS prevalence [28]. In 1990, the USA had the highest proportion of DALY among G20 countries, with males being predominantly affected. By 2019, South Africa and India had the highest proportion, with more women being affected. In South Africa, the higher risk of HIV infection among women compared to men is linked to factors such as poverty, low education levels, transactional sex, and legal barriers to health services for women [18]. Due to these factors, women in sub-Saharan Africa have an estimated HIV prevalence up to 2.7 times higher than men in the region [18]. Therefore, the gender disparity in DALY among G20 is gradually narrowing. In the past 30 years, China has seen significant changes in AIDS transmission and affected populations. Initially, transmission was mainly through intravenous drug use, but now it's predominantly sexual. About 95% of new HIV infections are sexually transmitted, with the proportion of men who have sex with men (MSM) increasing from 0.3 in 2006 to 28.4% in 2015, further widening the gender disparity in AIDS prevalence [29].

In term of age group, our study indicated that the 30–49 age group exhibited the highest ASDR both in China and the G20. This finding has been supported by other studies [30–32]. Although the ASDR in this age group decreased since 2007, it continued to increase in China, particularly within the 30–44 age group. This can be attributed to the fact that this age group was the most sexually active period and also a high-risk age group for drug use. Furthermore, individuals within this age group demonstrated high population mobility and had extensive social networks, but there was a lack of awareness about AIDS and the adoption of unhealthy lifestyle habits. Consequently, this age group bears a significantly higher burden of AIDS compared to other age groups, and the trend of increase was rapid.

In terms of future trends, our study projects that China's DALY will continue to rise over the next 20 years, indicating ongoing challenges in AIDS prevention and control. Although China has established and expanded sentinel surveillance, the number of newly diagnosed HIV infections continues to increase. Furthermore, the percentage of undiagnosed infections remains as high as 30%, which could pose significant challenges to future prevention and control efforts [24]. Additionally, changes in transmission methods and the increasing proportion of MSM have a profound impact on the future prevention and control of HIV/AIDS. Moreover, the developments of the internet and social progress have led to a more dispersed source of HIV transmission and more concealed pathways, further increasing the difficulty of prevention and control. Lastly, the complications resulting from long-term antiretroviral therapy and the associated personal and family burdens also affect the effectiveness of HIV/AIDS prevention and control.

The study offers key insights for AIDS prevention and control in China. First, China could adopt strategies from the United States for raising AIDS awareness among youth. This involves using leveraging digital media and social platforms to create interactive and engaging HIV/AIDS information [14,15]. Integrating Chinese cultural and social aspects is critical in developing localised educational methods and materials. Second, precise interventions for high-risk groups, especially the MSM (Men who have Sex with Men) community, are essential. This strategy should encompass the provision of preventive tools like condoms and needles, along with regular health check-ups and counselling to mitigate infection risks. Additionally, enhancing sexual health education and AIDS awareness among the 30–49 age group is vital, utilising social networks and community initiatives to disseminate preventive information effectively. Lastly, enhancing testing capabilities, along with the diagnostic and treatment processes, is essential for effective AIDS control in China. Improving service coverage and quality and shortening the HIV window period are important to ensure the timely detection of new cases and interrupt the transmission chains.

There are limitations in our study. The GBD 2019 offers a substantial framework for assessing the burden of AIDS. However, it faces challenges related to data quality and completeness, particularly in lower-income regions and remote areas. These limitations could potentially impact the accuracy of analytical findings regarding AIDS, highlighting the need for cautious interpretation and consideration of data constraints in these specific contexts. Additionally, due to the hidden nature of HIV/AIDS, some cases may go unreported, resulting in an underestimation of the disease burden and introducing biases into the analysis results. Apart from the GBD database, there are other databases that provide data about HIV/AIDS. However, analysing a single database alone does not provide a comprehensive understanding of the disease's trends and changes. To address current limitations, our future research strategy involves a methodological expansion. We aim to integrate various databases to achieve a deeper and more comprehensive understanding of HIV/AIDS trends. This approach will enhance the generalisability and robustness of our findings, providing a more solid base for conclusions and recommendations.

## CONCLUSIONS

Despite the significant efforts made by China and G20 countries in AIDS prevention and control, HIV/AIDS continues to pose a significant disease burden on China and the G20 countries. In addition, the ASMR and ASDR was predicted to increase in the next 20 years. Therefore, to address the challenges of future HIV/AIDS prevention and control, China needs to further strengthen HIV/AIDS prevention, monitoring, and treatment, as well as developing targeted policies and measures for prevention and control.

## Additional material


Online Supplementary Document
