# Supplementary material for: The trend of AIDS in China: A prediction and comparative analysis with G20 countries based on the Global Burden of Disease Study 2019
Source: J Glob Health. 2024 Mar 1;14:04029. doi: 10.7189/jogh.14.04029 (PMC10906135; doi:10.7189/jogh.14.04029)
Supplement: Online Supplementary Document [file jogh-14-04029-s001.pdf]

Group	Age	Incidence	Mortality	DALYs	MIR
Global	ALL	-1.40	2.00	1.50	3.40
Global	15 to 19	-1.60	3.70	3.50	5.30
Global	20 to 24	-1.40	0.80	0.90	2.30
Global	25 to 29	-1.20	0.40	0.50	1.60
Global	30 to 34	-1.10	1.10	1.20	2.20
Global	35 to 39	-1.20	2.20	2.30	3.40
Global	40 to 44	-1.70	2.80	2.90	4.50
Global	45 to 49	-2.50	2.70	2.90	5.40
Global	50 to 54	-2.40	2.90	3.00	5.50
Global	55 to 59	-1.20	2.90	3.20	4.30
Global	60 to 64	-0.60	2.90	3.20	3.70
Global	65 to 69	-1.00	2.60	2.90	3.60
Global	70 to 74	-2.60	1.90	2.40	4.90
Global	75 to 79	-3.20	2.20	2.50	5.40
G20	ALL	3.30	4.50	4.30	1.10
G20	15 to 19	3.50	8.20	7.90	4.70
G20	20 to 24	2.50	4.90	4.80	2.30
G20	25 to 29	3.50	2.90	3.00	-0.60
G20	30 to 34	3.60	3.00	3.10	-0.60
G20	35 to 39	3.80	3.90	4.00	0.10
G20	40 to 44	3.00	4.00	4.20	1.20
G20	45 to 49	2.50	4.50	4.70	2.00
G20	50 to 54	2.30	5.40	5.50	2.90
G20	55 to 59	4.20	5.30	5.50	0.90
G20	60 to 64	4.90	5.00	5.40	0.30
G20	65 to 69	5.90	4.60	5.20	-1.10
G20	70 to 74	4.00	8.10	8.40	4.10
G20	75 to 79	-1.40	7.50	6.60	7.30
China	ALL	3.20	7.90	6.80	4.60
China	15 to 19	7.10	8.20	7.90	1.40
China	20 to 24	6.40	6.80	6.50	0.60
China	25 to 29	3.70	6.40	6.20	3.00
China	30 to 34	2.80	6.90	6.80	4.60
China	35 to 39	1.60	7.30	7.10	6.30
China	40 to 44	1.20	6.50	6.50	6.10
China	45 to 49	1.50	7.20	7.10	5.70
China	50 to 54	2.20	7.00	8.20	5.40
China	55 to 59	2.20	6.50	6.50	4.50
China	60 to 64	2.90	6.00	6.10	3.10
China	65 to 69	5.10	7.70	7.60	2.80
China	70 to 74	3.00	8.80	8.70	6.20
China	75 to 79	-3.60	6.30	5.50	10.30

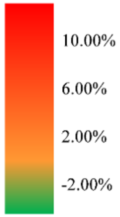

Figure S1. The AAPC of ASIR, ASMR, ASDR and ASR of MIR across different age groups in the world, G20 countries and China.

Table S1. The incidence cases, ASIR, and temporal trend of AIDS from 1990 to 2019 in China and G20

Characteristics		1990		2019		1990–2019
		Incidence cases	ASIR per 100,000	Incidence cases	ASIR per 100,000	AAPC
		No.×10^3 (95% UI)	No. (95% UI)	No.×10^3 (95% UI)	No. (95% UI)	No. (95% UI)
China	**Total**	9.67(6.31-17.67)	0.82(0.53-1.49)	31.88(15.36-51.46)	2.24(1.08-3.62)	3.20(1.80-4.70)
	**Male**	6.48(4.22-11.93)	1.06(0.69-1.96)	24.84(11.95-40.04)	3.43(1.65-5.52)	3.80(2.60-4.90)
	**Female**	3.19(2.10-5.76)	0.56(0.37-1.00)	7.03(3.43-11.15)	1.01(0.49-1.60)	2.10(1.40-2.90)
	**Age**					
	15 to 19	0.59(0.39-0.87)	0.46(0.31-0.69)	2.49(0.97-4.85)	3.32(1.29-6.46)	7.10(5.50-8.80)
	20 to 24	1.59(0.93-2.62)	1.20(0.70-1.98)	6.01(2.72-10.86)	7.35(3.32-13.27)	6.40(5.30-7.50)
	25 to 29	1.61(0.99-3.11)	1.46(0.90-2.82)	4.97(2.07-10.75)	4.49(1.87-9.71)	3.70(2.60-4.80)
	30 to 34	1.10(0.64-2.20)	1.25(0.72-2.49)	3.76(1.35-6.35)	2.91(1.05-4.92)	2.80(1.90-3.70)
	35 to 39	1.12(0.64-2.35)	1.22(0.70-2.57)	1.98(0.64-3.96)	1.96(0.64-3.93)	1.60(0.50-2.60)
	40 to 44	0.68(0.38-1.50)	1.01(0.57-2.24)	1.47(0.53-2.16)	1.45(0.52-2.13)	1.20(0.40-2.00)
	45 to 49	0.39(0.23-0.86)	0.75(0.44-1.66)	1.46(0.49-2.47)	1.20(0.41-2.03)	1.50(0.30-2.70)
	50 to 54	0.35(0.22-0.71)	0.73(0.46-1.49)	1.81(0.64-2.58)	1.45(0.51-2.06)	2.20(0.60-3.80)
	55 to 59	0.35(0.22-0.69)	0.81(0.51-1.59)	1.53(0.61-2.19)	1.61(0.64-2.31)	2.20(0.50-4.00)
	60 to 64	0.35(0.22-0.71)	0.99(0.61-2.01)	1.98(0.85-2.85)	2.52(1.08-3.63)	2.90(1.80-4.00)
	65 to 69	0.18(0.11-0.31)	0.68(0.41-1.13)	2.07(0.91-3.14)	2.94(1.29-4.46)	5.10(3.80-6.50)
	70 to 74	0.14(0.08-0.21)	0.73(0.41-1.10)	0.88(0.41-1.37)	1.84(0.87-2.87)	3.00(1.40-4.60)
	75 to 79	0.52(0.20-1.44)	4.58(1.75-12.62)	0.64(0.27-0.97)	2.16(0.90-3.26)	-3.60(-6.90--0.10)
	**Total**	249.68(211.76-294.10)	6.74(5.72-7.94)	801.73(690.08-917.72)	16.48(14.19-18.87)	3.30(2.70-3.80)
	**Male**	162.38(135.51-193.94)	8.68(7.25-10.37)	444.69(379.82-511.59)	18.17(15.52-20.90)	2.70(2.20-3.10)

	**Female**	87.30(73.07-103.07)	4.77(3.99-5.63)	357.03(300.01-425.24)	14.78(12.42-17.60)	3.90(3.50-4.30)
	**Age**					
	15 to 19	20.78(15.88-26.54)	5.91(4.51-7.54)	52.81(44.26-62.49)	15.30(12.83-18.11)	3.50(2.80-4.10)
	20 to 24	48.43(37.72-61.81)	14.00(10.90-17.87)	95.84(81.41-113.18)	27.40(23.28-32.36)	2.50(2.00-2.90)
	25 to 29	55.43(46.32-66.37)	17.68(14.78-21.17)	168.46(141.46-204.34)	44.98(37.77-54.56)	3.50(2.90-4.10)
	30 to 34	43.77(36.90-52.33)	15.86(13.37-18.96)	168.04(143.15-195.07)	43.03(36.65-49.95)	3.60(3.00-4.20)
	35 to 39	30.38(25.44-36.36)	11.61(9.72-13.90)	113.64(83.13-154.71)	32.23(23.58-43.88)	3.80(2.90-4.70)
	40 to 44	17.25(14.32-20.68)	8.00(6.64-9.60)	56.90(42.67-73.28)	17.13(12.84-22.06)	3.00(2.20-3.70)
	45 to 49	9.21(7.87-10.83)	5.32(4.55-6.26)	34.85(24.79-48.10)	10.40(7.40-14.36)	2.50(1.90-3.00)
	50 to 54	6.94(6.13-7.81)	4.36(3.85-4.90)	26.11(18.79-34.47)	8.20(5.91-10.83)	2.30(1.80-2.80)
	55 to 59	4.35(3.83-4.99)	3.08(2.71-3.53)	26.24(16.75-36.53)	9.67(6.17-13.47)	4.20(2.90-5.50)
	60 to 64	2.80(2.44-3.25)	2.27(1.98-2.64)	19.96(13.90-25.84)	8.56(5.96-11.09)	4.90(4.50-5.40)
	65 to 69	1.35(1.13-1.60)	1.40(1.17-1.67)	13.81(10.35-17.27)	6.92(5.18-8.65)	5.90(5.20-6.60)
**G20**	70 to 74	0.84(0.71-0.98)	1.29(1.09-1.50)	5.67(4.47-7.13)	3.86(3.04-4.85)	4.00(3.30-4.70)
	75 to 79	1.41(0.77-3.10)	2.91(1.58-6.39)	2.17(1.62-2.76)	2.18(1.62-2.76)	-1.40(-2.90-0.10)
	**Regions**					
	Argentina	5.16(3.56-6.99)	15.57(10.75-21.12)	11.63(5.96-19.37)	25.79(13.20-42.93)	1.80(1.20-2.30)
	Australia	1.16(0.95-1.36)	6.87(5.64-8.05)	1.49(0.87-2.20)	6.05(3.54-8.97)	-0.50(-1.30-0.30)
	Brazil	26.95(21.95-31.76)	18.11(14.75-21.34)	64.76(50.31-78.78)	29.89(23.22-36.36)	1.80(1.10-2.60)
	Canada	2.79(1.47-4.05)	10.25(5.40-14.85)	3.32(1.58-5.25)	9.10(4.32-14.37)	-0.40(-1.30-0.50)
	China	9.67(6.31-17.67)	0.82(0.53-1.49)	31.88(15.36-51.46)	2.24(1.08-3.62)	3.20(1.80-4.70)
	European Union	27.70(22.63-39.16)	5.79(4.73-8.18)	26.17(20.26-32.33)	5.08(3.94-6.28)	-0.50(-0.90-0.00)
	France	3.63(3.20-4.05)	6.28(5.54-7.02)	2.57(1.51-3.94)	3.88(2.29-5.95)	-1.50(-2.00--1.00)
	Germany	2.99(2.46-3.69)	3.74(3.08-4.61)	2.31(1.48-3.46)	2.72(1.74-4.07)	-1.00(-2.10-0.10)
	India	27.73(11.04-56.54)	3.24(1.29-6.61)	73.45(44.49-115.56)	5.28(3.20-8.31)	1.60(0.60-2.60)
	Indonesia	2.75(0.00-4.97)	1.48(0.00-2.68)	14.03(11.52-18.31)	5.41(4.44-7.06)	4.30(2.30-6.30)

Italy	6.14(1.88-17.00)	10.81(3.30-29.94)	3.85(2.39-6.15)	6.38(3.96-10.19)	-2.50(-3.80--1.10)
Japan	0.65(0.34-1.07)	0.51(0.27-0.85)	2.72(1.22-4.20)	2.13(0.95-3.29)	5.30(4.40-6.20)
Mexico	17.21(12.17-23.58)	20.13(14.23-27.58)	16.89(13.38-21.31)	13.52(10.71-17.05)	-1.20(-1.50--0.90)
Republic of Korea	0.34(0.00-1.09)	0.78(0.00-2.46)	1.26(0.32-2.41)	2.36(0.60-4.51)	5.60(2.10-9.30)
Russian Federation	6.75(5.18-8.30)	4.47(3.43-5.49)	118.63(95.42-150.27)	80.85(65.03-102.42)	10.60(7.80-13.50)
Saudi Arabia	0.23(0.12-0.51)	1.43(0.73-3.19)	1.04(0.39-2.47)	2.90(1.10-6.91)	2.50(2.10-2.90)
South Africa	61.51(48.95-76.88)	167.01(132.92-208.75)	366.88(286.12-464.31)	659.99(514.71-835.27)	4.90(4.50-5.20)
Turkey	0.04(0.00-0.11)	0.06(0.00-0.18)	0.44(0.32-0.62)	0.55(0.39-0.77)	6.70(6.00-7.50)
United Kingdom	2.11(1.43-3.18)	3.67(2.49-5.54)	5.46(3.14-7.96)	8.12(4.67-11.84)	2.80(2.00-3.60)
United States of America	59.04(36.12-83.38)	23.28(14.24-32.88)	67.13(28.61-104.89)	20.47(8.72-31.98)	-0.40(-1.80-1.00)

Table S2 The mortality cases, ASMR, and temporal trend of AIDS from 1990 to 2019 in China and G20

Characteristics		1990		2019		1990–2019
		**Mortality cases**	**ASMR per 100,000**	**Mortality cases**	**ASMR per 100,000**	**AAPC**
		**No.×10^3 (95% UI)**	**No. (95% UI)**	**No.×10^3 (95% UI)**	**No. (95% UI)**	**No. (95% UI)**
**China**	**Total**	2.70(0.73-3.89)	0.23(0.06-0.33)	31.75(25.78-37.45)	2.23(1.81-2.63)	7.90(6.60-9.20)
	**Male**	1.79(0.45-2.63)	0.29(0.07-0.43)	23.79(19.42-27.95)	3.28(2.68-3.86)	8.30(7.10-9.50)
	**Female**	0.91(0.27-1.27)	0.16(0.05-0.22)	7.95(6.26-9.54)	1.14(0.90-1.37)	6.60(5.10-8.00)
	**Age**					
	15 to 19	0.03(0.01-0.05)	0.02(0.01-0.04)	0.19(0.14-0.23)	0.25(0.18-0.30)	8.20(7.50-8.90)
	20 to 24	0.14(0.04-0.22)	0.10(0.03-0.17)	0.58(0.50-0.62)	0.70(0.61-0.76)	6.80(6.20-7.40)
	25 to 29	0.29(0.06-0.48)	0.26(0.05-0.43)	1.83(1.54-2.11)	1.65(1.39-1.91)	6.40(5.70-7.10)
	30 to 34	0.29(0.06-0.48)	0.33(0.07-0.54)	3.29(2.73-3.90)	2.55(2.12-3.02)	6.90(6.20-7.70)
	35 to 39	0.34(0.06-0.57)	0.37(0.06-0.62)	3.20(2.36-4.39)	3.17(2.34-4.35)	7.30(6.30-8.30)
	40 to 44	0.26(0.04-0.44)	0.39(0.06-0.65)	3.33(2.52-4.16)	3.28(2.48-4.09)	6.50(5.40-7.50)
	45 to 49	0.20(0.03-0.32)	0.39(0.06-0.63)	4.28(3.09-5.75)	3.53(2.55-4.74)	7.20(5.80-8.50)
	50 to 54	0.15(0.02-0.23)	0.32(0.04-0.49)	4.02(3.02-5.10)	3.22(2.41-4.08)	7.00(5.70-8.40)
	55 to 59	0.14(0.02-0.21)	0.33(0.04-0.49)	2.31(1.81-2.80)	2.43(1.91-2.95)	6.50(5.20-7.70)
	60 to 64	0.13(0.02-0.20)	0.38(0.05-0.57)	2.01(1.65-2.35)	2.56(2.10-2.99)	6.00(4.80-7.20)
	65 to 69	0.10(0.01-0.15)	0.36(0.05-0.55)	2.52(2.06-2.94)	3.58(2.92-4.17)	7.70(5.80-9.70)
	70 to 74	0.05(0.01-0.08)	0.27(0.04-0.44)	1.72(1.35-2.05)	3.59(2.83-4.28)	8.80(7.70-9.90)
	75 to 79	0.06(0.03-0.09)	0.56(0.22-0.80)	0.95(0.73-1.14)	3.19(2.46-3.82)	6.30(3.80-8.80)
	**Total**	60.47(58.08-63.23)	1.63(1.57-1.71)	280.86(260.66-313.79)	5.77(5.36-6.45)	4.50(3.50-5.50)
	**Male**	49.08(47.53-50.67)	2.62(2.54-2.71)	157.98(144.54-179.46)	6.45(5.90-7.33)	3.20(2.10-4.30)

	**Female**	11.39(10.46-12.64)	0.62(0.57-0.69)	122.88(109.41-144.44)	5.09(4.53-5.98)	7.60(6.60-8.60)
	**Age**					
	15 to 19	0.60(0.55-0.72)	0.17(0.16-0.20)	5.79(4.44-7.62)	1.68(1.29-2.21)	8.20(7.50-9.00)
	20 to 24	2.45(2.28-2.70)	0.71(0.66-0.78)	9.71(7.19-13.38)	2.78(2.06-3.82)	4.90(4.20-5.60)
	25 to 29	7.85(7.46-8.35)	2.50(2.38-2.66)	21.55(14.03-31.07)	5.75(3.75-8.29)	2.90(2.20-3.60)
	30 to 34	11.50(11.11-12.01)	4.17(4.02-4.35)	38.28(29.80-49.57)	9.80(7.63-12.69)	3.00(1.80-4.20)
	35 to 39	11.23(10.78-11.79)	4.29(4.12-4.51)	44.53(34.63-56.99)	12.63(9.82-16.17)	3.90(2.70-5.00)
	40 to 44	8.64(8.31-9.00)	4.01(3.85-4.18)	40.91(35.96-46.51)	12.32(10.83-14.00)	4.00(2.80-5.30)
	45 to 49	5.21(4.97-5.46)	3.01(2.87-3.15)	35.74(29.75-42.83)	10.67(8.88-12.78)	4.50(3.40-5.60)
	50 to 54	3.11(2.94-3.27)	1.95(1.85-2.05)	27.76(24.22-32.80)	8.72(7.61-10.31)	5.40(4.50-6.20)
	55 to 59	2.18(2.04-2.30)	1.55(1.45-1.63)	17.80(15.68-21.05)	6.56(5.78-7.76)	5.30(3.90-6.60)
	60 to 64	1.52(1.40-1.62)	1.24(1.14-1.31)	11.68(10.26-13.72)	5.01(4.40-5.89)	5.00(3.90-6.20)
	65 to 69	1.07(0.98-1.14)	1.11(1.02-1.18)	8.26(7.33-9.54)	4.14(3.67-4.78)	4.60(3.50-5.70)
**G20**	70 to 74	0.19(0.15-0.24)	0.30(0.22-0.36)	4.17(3.58-4.94)	2.84(2.43-3.36)	8.10(7.60-8.60)
	75 to 79	0.15(0.10-0.18)	0.30(0.21-0.37)	2.22(1.91-2.60)	2.22(1.91-2.61)	7.50(6.20-8.80)
	**Regions**					
	Argentina	0.56(0.55-0.57)	1.69(1.66-1.72)	1.78(1.74-1.82)	3.94(3.85-4.02)	3.10(2.40-3.70)
	Australia	0.38(0.37-0.39)	2.25(2.18-2.31)	0.07(0.07-0.07)	0.29(0.28-0.30)	-7.60(-10.50--4.60)
	Brazil	8.07(7.93-8.20)	5.42(5.33-5.51)	15.56(15.30-15.84)	7.18(7.06-7.31)	0.90(0.30-1.50)
	Canada	0.80(0.77-0.83)	2.94(2.84-3.03)	0.25(0.24-0.27)	0.69(0.66-0.73)	-4.70(-6.70--2.70)
	China	2.70(0.73-3.89)	0.23(0.06-0.33)	31.75(25.78-37.45)	2.23(1.81-2.63)	7.90(6.60-9.20)
	European Union	10.54(10.44-10.65)	2.20(2.18-2.22)	3.73(3.67-3.79)	0.72(0.71-0.74)	-4.20(-5.50--3.00)
	France	3.05(2.99-3.12)	5.28(5.17-5.40)	0.46(0.43-0.48)	0.69(0.66-0.73)	-7.20(-8.90--5.50)
	Germany	1.68(1.64-1.72)	2.10(2.05-2.15)	0.42(0.40-0.44)	0.50(0.47-0.52)	-4.90(-5.80--4.00)
	India	1.14(0.83-1.76)	0.13(0.10-0.21)	46.30(40.99-53.46)	3.33(2.95-3.84)	11.80(10.70-12.80)
	Indonesia	0.01(0.00-0.02)	0.01(0.00-0.01)	5.60(4.49-7.43)	2.16(1.73-2.86)	21.20(19.60-22.80)

Italy	1.86(1.83-1.90)	3.28(3.22-3.34)	0.62(0.61-0.63)	1.03(1.01-1.05)	-4.40(-5.70--3.10)
Japan	0.07(0.07-0.07)	0.05(0.05-0.06)	0.16(0.16-0.16)	0.13(0.12-0.13)	2.90(1.10-4.70)
Mexico	2.06(2.03-2.09)	2.41(2.38-2.44)	5.03(4.98-5.09)	4.03(3.98-4.07)	1.70(1.20-2.20)
Republic of Korea	0.05(0.05-0.05)	0.12(0.11-0.12)	0.14(0.13-0.15)	0.26(0.24-0.28)	2.00(0.00-4.10)
Russian Federation	3.38(3.35-3.40)	2.24(2.22-2.25)	18.68(18.48-18.88)	12.73(12.60-12.87)	6.30(4.80-7.80)
Saudi Arabia	0.07(0.04-0.12)	0.43(0.25-0.75)	0.68(0.28-1.81)	1.91(0.77-5.07)	5.30(5.20-5.40)
South Africa	2.85(1.98-4.48)	7.74(5.39-12.17)	143.85(123.86-177.38)	258.78(222.82-319.10)	12.90(11.00-14.80)
Turkey	0.01(0.00-0.01)	0.01(0.00-0.02)	0.23(0.18-0.26)	0.28(0.22-0.33)	11.00(10.40-11.60)
United Kingdom	0.46(0.45-0.46)	0.80(0.79-0.80)	0.23(0.23-0.23)	0.35(0.34-0.35)	-3.20(-5.80--0.50)
United States of America	27.79(27.55-28.02)	10.96(10.86-11.05)	7.05(6.97-7.13)	2.15(2.13-2.17)	-5.70(-6.40--5.00)

Table S3. The incidence cases, ASIR, and temporal trend of AIDS from 1990 to 2019 worldwide

Characteristics	1990		2019		1990–2019
	Incidence cases	ASIR per 100,000	Incidence cases	ASIR per 100,000	AAPC
	No.×10^3 (95% UI)	No. (95% UI)	No.×10^3 (95% UI)	No. (95% UI)	No. (95% UI)
**Total**	2057.71(1708.17-2430.84)	38.46(31.93-45.44)	1989.28(1760.91-2259.35)	25.71(22.76-29.20)	-1.40(-1.60--1.20)
**Male**	919.82(780.54-1063.67)	34.15(28.98-39.49)	990.70(877.73-1110.29)	25.53(22.62-28.61)	-1.10(-1.30--0.80)
**Female**	1137.89(916.51-1371.01)	42.84(34.51-51.62)	998.58(861.84-1165.36)	25.89(22.35-30.22)	-1.80(-2.00--1.60)
**Age**					
15 to 19	242.83(200.45-282.57)	46.73(38.58-54.38)	183.99(161.22-214.80)	29.70(26.02-34.67)	-1.60(-1.90--1.30)
20 to 24	290.81(246.77-335.32)	59.03(50.09-68.06)	238.45(210.94-271.29)	39.73(35.15-45.20)	-1.40(-1.60--1.20)
25 to 29	429.03(351.29-511.86)	96.88(79.33-115.59)	416.63(363.17-480.19)	68.81(59.98-79.31)	-1.20(-1.40--1.00)
30 to 34	307.81(251.79-367.45)	79.82(65.29-95.29)	351.85(306.67-401.86)	58.47(50.96-66.78)	-1.10(-1.30--0.90)
35 to 39	232.31(184.52-284.06)	65.86(52.31-80.53)	254.35(199.40-312.29)	47.02(36.86-57.73)	-1.20(-1.40--0.90)
40 to 44	132.07(104.55-162.40)	46.13(36.52-56.73)	138.35(109.41-172.60)	28.04(22.17-34.98)	-1.70(-1.90--1.50)
45 to 49	90.27(71.41-112.08)	38.84(30.73-48.23)	88.71(68.93-114.37)	18.72(14.55-24.14)	-2.50(-2.70--2.30)
50 to 54	59.10(47.54-72.26)	27.80(22.36-33.99)	60.32(46.53-76.17)	13.81(10.65-17.44)	-2.40(-2.60--2.20)
55 to 59	36.67(29.40-44.95)	19.78(15.85-24.24)	51.87(36.05-69.63)	13.98(9.72-18.77)	-1.20(-1.40--1.00)
60 to 64	23.14(18.04-28.84)	14.40(11.23-17.95)	38.02(27.40-49.43)	12.17(8.77-15.82)	-0.60(-0.80--0.50)
65 to 69	15.41(11.61-19.75)	12.48(9.40-15.99)	24.30(18.94-30.72)	9.40(7.32-11.88)	-1.00(-1.30--0.80)
70 to 74	11.13(8.41-14.21)	13.16(9.96-16.81)	11.14(9.15-13.63)	5.96(4.89-7.29)	-2.60(-3.20--2.00)
75 to 79	4.60(3.73-6.62)	7.50(6.08-10.80)	3.86(3.07-4.86)	3.04(2.41-3.83)	-3.20(-3.70--2.70)
**SDI regions**					
Low SDI	1051.75(798.84-1330.43)	199.14(151.26-251.91)	533.28(408.43-696.99)	47.25(36.19-61.75)	-4.90(-5.10--4.80)

Low-middle SDI	618.02(460.94-790.46)	54.71(40.80-69.97)	448.21(371.99-547.49)	25.41(21.09-31.04)	-2.70(-3.00--2.30)
Middle SDI	235.24(199.08-273.55)	13.70(11.60-15.93)	679.98(598.85-776.94)	28.37(24.99-32.42)	2.40(2.00-2.90)
High-middle SDI	70.51(56.92-89.59)	6.13(4.95-7.79)	232.20(197.63-279.23)	16.23(13.82-19.52)	3.40(3.10-3.80)
High SDI	79.89(54.33-107.63)	9.72(6.61-13.09)	94.03(50.41-137.75)	9.28(4.97-13.59)	-0.10(-0.90-0.70)

Table S4. The mortality cases, ASMR, and temporal trend of AIDS from 1990 to 2019 worldwide

Characteristics	1990		2019		1990–2019
	**Mortality cases**	**ASMR per 100,000**	**Mortality cases**	**ASMR per 100,000**	**AAPC**
	**No.×10^3 (95% UI)**	**No. (95% UI)**	**No.×10^3 (95% UI)**	**No. (95% UI)**	**No. (95% UI)**
**Total**	336.39(255.68-452.19)	6.29(4.78-8.45)	863.84(786.07-996.04)	11.16(10.16-12.87)	2.00(1.50-2.40)
**Male**	171.12(135.16-220.59)	6.35(5.02-8.19)	435.39(398.81-499.87)	11.22(10.28-12.88)	2.00(1.40-2.50)
**Female**	165.26(120.53-229.65)	6.22(4.54-8.65)	428.44(382.91-501.68)	11.11(9.93-13.01)	1.90(1.40-2.40)
**Age**					
15 to 19	7.96(5.06-13.27)	1.53(0.97-2.55)	27.66(17.30-40.64)	4.47(2.79-6.56)	3.70(3.20-4.20)
20 to 24	22.15(14.82-34.76)	4.50(3.01-7.05)	34.87(24.87-49.12)	5.81(4.14-8.18)	0.80(0.50-1.20)
25 to 29	40.97(24.85-62.59)	9.25(5.61-14.13)	64.18(40.58-94.37)	10.60(6.70-15.59)	0.40(-0.10-0.90)
30 to 34	47.49(32.18-69.15)	12.32(8.35-17.93)	102.75(76.95-137.81)	17.07(12.79-22.90)	1.10(0.50-1.70)
35 to 39	43.16(27.88-65.00)	12.24(7.90-18.43)	125.61(95.19-165.13)	23.22(17.60-30.53)	2.20(1.90-2.50)
40 to 44	32.18(21.64-45.28)	11.24(7.56-15.82)	123.86(109.68-140.93)	25.10(22.23-28.56)	2.80(2.40-3.20)
45 to 49	23.86(16.10-33.61)	10.27(6.93-14.46)	109.14(92.64-127.11)	23.03(19.55-26.83)	2.70(2.40-3.00)
50 to 54	16.88(11.82-22.88)	7.94(5.56-10.76)	81.01(72.22-94.00)	18.55(16.53-21.52)	2.90(2.50-3.20)
55 to 59	10.58(7.52-14.15)	5.71(4.05-7.63)	50.79(45.21-59.55)	13.69(12.19-16.05)	2.90(2.60-3.30)
60 to 64	6.69(4.79-8.95)	4.16(2.98-5.57)	31.17(27.57-36.59)	9.97(8.82-11.71)	2.90(2.60-3.20)
65 to 69	4.39(3.15-5.90)	3.55(2.55-4.78)	19.44(17.29-22.51)	7.52(6.68-8.70)	2.60(2.00-3.30)
70 to 74	2.47(1.60-3.57)	2.93(1.89-4.22)	9.85(8.65-11.52)	5.26(4.62-6.16)	1.90(1.30-2.60)
75 to 79	1.24(0.84-1.73)	2.03(1.37-2.81)	4.91(4.30-5.79)	3.86(3.38-4.56)	2.20(1.60-2.70)
**SDI regions**					
Low SDI	204.76(144.46-285.68)	38.77(27.35-54.09)	267.80(233.31-324.05)	23.73(20.67-28.71)	-1.80(-2.30--1.30)

Low-middle SDI	60.61(41.88-91.70)	5.37(3.71-8.12)	255.79(228.67-295.04)	14.50(12.96-16.73)	3.40(2.70-4.10)
Middle SDI	17.18(13.96-21.91)	1.00(0.81-1.28)	278.03(252.27-325.77)	11.60(10.53-13.59)	8.80(7.60-10.10)
High-middle SDI	17.53(16.95-18.49)	1.52(1.47-1.61)	50.80(49.11-53.14)	3.55(3.43-3.71)	2.90(2.40-3.40)
High SDI	35.98(35.74-36.23)	4.38(4.35-4.41)	10.63(10.12-11.75)	1.05(1.00-1.16)	-4.70(-5.20--4.20)

Table S5. The DALY cases, ASDR, and temporal trend of AIDS from 1990 to 2019 worldwide

**Characteristics**	**1990**		**2019**		**1990–2019**
	**DALY cases**	**ASDR per 100,000**	**DALY cases**	**ASDR per 100,000**	**AAPC**
	**No.×10^3 (95% UI)**	**No. (95% UI)**	**No.×10^3 (95% UI)**	**No. (95% UI)**	**No. (95% UI)**
**Total**	20915.85(16525.43-27291.85)	390.96(308.90-510.14)	47632.18(42630.99-55650.04)	615.60(550.97-719.23)	1.50(1.20-1.90)
**Male**	10338.95(8409.66-13098.86)	383.81(312.19-486.27)	23345.78(21108.90-27140.35)	601.54(543.91-699.32)	1.60(1.20-2.00)
**Female**	10576.89(7998.63-14252.87)	398.21(301.14-536.61)	24286.40(21357.67-28616.85)	629.75(553.81-742.04)	1.50(1.00-2.00)
**Age**					
15 to 19	637.07(424.83-1011.50)	122.61(81.76-194.67)	2098.35(1347.42-3021.43)	338.69(217.49-487.69)	3.50(3.10-3.90)
20 to 24	1605.06(1107.88-2438.73)	325.78(224.87-495.00)	2558.75(1895.62-3500.75)	426.36(315.86-583.32)	0.90(0.50-1.30)
25 to 29	2710.64(1724.64-4069.96)	612.10(389.45-919.05)	4370.65(2975.26-6225.37)	721.86(491.40-1028.19)	0.50(0.00-0.90)
30 to 34	2860.58(1994.88-4089.97)	741.79(517.31-1060.60)	6396.59(4970.89-8383.98)	1063.03(826.10-1393.31)	1.20(0.60-1.90)
35 to 39	2369.81(1579.40-3495.44)	671.83(447.75-990.93)	7132.23(5610.98-9124.27)	1318.40(1037.20-1686.63)	2.30(1.70-2.90)
40 to 44	1593.16(1106.83-2204.83)	556.48(386.61-770.14)	6348.47(5700.95-7091.15)	1286.57(1155.34-1437.08)	2.90(2.50-3.30)
45 to 49	1059.35(724.90-1457.36)	455.81(311.91-627.06)	5014.35(4280.93-5823.12)	1058.32(903.53-1229.02)	2.90(2.60-3.20)
50 to 54	666.40(483.78-882.90)	313.47(227.56-415.30)	3320.86(2951.97-3874.28)	760.24(675.79-886.93)	3.00(2.70-3.30)
55 to 59	367.86(273.23-481.09)	198.40(147.36-259.47)	1863.16(1649.35-2188.42)	502.18(444.55-589.85)	3.20(2.60-3.90)
60 to 64	201.55(150.70-263.16)	125.47(93.81-163.82)	1005.50(884.41-1184.43)	321.72(282.98-378.97)	3.20(2.90-3.50)
65 to 69	111.73(83.42-146.54)	90.48(67.55-118.67)	538.52(472.79-632.28)	208.26(182.84-244.52)	2.90(2.50-3.40)
70 to 74	53.17(36.48-73.51)	62.92(43.16-86.98)	233.49(201.67-276.90)	124.80(107.80-148.01)	2.40(1.50-3.30)
75 to 79	22.70(16.98-29.72)	37.02(27.70-48.48)	96.27(82.62-115.48)	75.77(65.03-90.89)	2.50(2.00-2.90)
**SDI regions**					
Low SDI	12877.09(9534.04-17312.23)	2438.19(1805.21-3277.96)	15551.58(13229.66-18952.91)	1377.86(1172.14-1679.22)	-2.00(-2.70--1.40)

Low-middle SDI	3998.00(2885.37-5771.80)	353.92(255.42-510.94)	13950.23(12220.88-16341.38)	790.84(692.80-926.39)	2.70(2.10-3.30)
Middle SDI	1106.45(942.46-1348.93)	64.45(54.90-78.57)	14714.40(13273.53-17332.75)	613.98(553.86-723.23)	8.10(7.30-9.00)
High-middle SDI	999.44(964.76-1052.39)	86.88(83.86-91.48)	2752.78(2618.34-2938.58)	192.45(183.05-205.44)	2.70(2.20-3.20)
High SDI	1915.00(1858.59-1987.77)	232.97(226.10-241.82)	620.84(521.80-747.41)	61.26(51.49-73.75)	-5.00(-5.70--4.20)

Table S6 The MIR and temporal trend from 1990 to 2019 worldwide

**Characteristics**	**MIR in 1990**	**MIR in 2019**	**AAPC No. (95% UI)**
**Total**	0.16(0.11-0.22)	0.43(0.36-0.51)	3.40(3.10-3.70)
**Male**	0.19(0.14-0.25)	0.44(0.37-0.52)	3.00(2.60-3.50)
**Female**	0.15(0.09-0.21)	0.43(0.35-0.53)	3.80(3.50-4.10)
**Age**			
15 to 19	0.03(0.02-0.05)	0.15(0.08-0.22)	5.30(4.90-5.80)
20 to 24	0.08(0.04-0.11)	0.15(0.10-0.20)	2.30(2.00-2.60)
25 to 29	0.10(0.05-0.15)	0.15(0.09-0.22)	1.60(1.10-2.10)
30 to 34	0.15(0.09-0.23)	0.29(0.20-0.41)	2.20(1.50-2.90)
35 to 39	0.19(0.10-0.29)	0.49(0.34-0.68)	3.40(2.70-4.10)
40 to 44	0.24(0.16-0.37)	0.90(0.71-1.21)	4.50(4.00-5.00)
45 to 49	0.26(0.17-0.40)	1.23(0.93-1.74)	5.40(4.90-5.90)
50 to 54	0.29(0.19-0.42)	1.34(1.04-1.79)	5.50(5.30-5.70)
55 to 59	0.29(0.18-0.41)	0.98(0.70-1.47)	4.30(3.90-4.60)
60 to 64	0.29(0.19-0.41)	0.82(0.61-1.14)	3.70(3.40-4.00)
65 to 69	0.28(0.18-0.42)	0.80(0.62-1.06)	3.60(3.30-4.00)
70 to 74	0.22(0.13-0.34)	0.88(0.69-1.15)	4.90(4.60-5.20)
75 to 79	0.27(0.16-0.46)	1.27(0.97-1.71)	5.40(4.70-6.10)
**SDI regions**			
Low SDI	0.19(0.12-0.29)	0.50(0.37-0.73)	3.30(3.00-3.70)
Low-middle SDI	0.10(0.05-0.15)	0.57(0.45-0.73)	6.20(5.90-6.50)

Middle SDI	0.07(0.05-0.10)	0.41(0.34-0.49)	6.20(5.50-7.00)
High-middle SDI	0.25(0.20-0.32)	0.22(0.18-0.27)	-0.50(-1.90-0.90)
High SDI	0.45(0.34-0.68)	0.11(0.07-0.22)	-5.30(-7.00--3.50)

Table S7. The prediction of incidence in next 20 years in China and G20

Characteristics		2030		2040	
		**Incidence cases No.×10³ (95% UI)**	**ASIR per 100,000 No. (95% UI)**	**Incidence cases No.×10³ (95% UI)**	**ASIR per 100,000 No. (95% UI)**
**China**	**Total**	29.59(0.00-101.03)	1.99(0.00-7.51)	27.52(0.00-210.25)	1.76(0.00-15.89)
	**Male**	22.23(0.00-85.49)	3.12(0.00-10.33)	19.81(0.00-185.91)	2.83(0.00-21.29)
	**Female**	9.09(1.54-16.63)	1.41(0.23-2.58)	8.72(0.76-16.68)	1.34(0.11-2.57)
	**Age**				
	15 to 19	2.30(0.00-4.98)	4.12(2.69-5.55)	2.28(0.00-6.92)	5.03(2.91-7.15)
	20 to 24	4.92(0.00-12.46)	7.84(2.89-12.79)	3.91(0.00-22.90)	7.88(0.00-16.02)
	25 to 29	2.80(0.00-16.25)	5.98(0.00-16.63)	0.82(0.00-35.24)	7.33(0.00-34.58)
	30 to 34	4.47(1.12-7.83)	3.51(0.65-6.37)	3.97(0.17-7.76)	3.37(0.38-6.36)
	35 to 39	3.70(0.00-7.75)	3.47(0.94-5.99)	3.16(0.00-7.46)	2.83(0.03-5.62)
	40 to 44	2.88(0.44-5.31)	2.88(0.61-5.15)	2.65(0.00-5.35)	2.37(0.00-4.86)
	45 to 49	1.37(0.00-8.81)	2.31(0.59-4.04)	1.30(0.00-21.11)	1.89(0.00-3.79)
	50 to 54	1.91(0.00-4.70)	2.67(0.00-5.90)	1.92(0.00-4.82)	2.34(0.00-5.74)
	55 to 59	2.44(0.00-6.25)	3.45(0.12-6.77)	2.08(0.00-6.73)	2.73(0.00-6.39)
	60 to 64	0.79(0.00-5.15)	2.14(0.00-15.11)	0.00(0.00-10.85)	1.79(0.00-36.47)
	65 to 69	1.55(0.00-3.83)	1.09(0.00-13.59)	1.70(0.00-4.69)	0.00(0.00-33.04)
	70 to 74	1.72(0.00-4.41)	0.68(0.00-12.29)	1.41(0.00-4.22)	0.00(0.00-12.31)
	75 to 79	0.96(0.00-7.15)	9.61(0.00-21.82)	0.97(0.00-9.48)	6.55(0.00-19.78)
	**Total**	908.79(521.10-1296.47)	22.65(13.92-31.37)	806.82(321.24-1292.40)	17.42(6.46-28.39)
	**Male**	367.06(0.00-1349.34)	22.06(14.14-29.99)	310.76(0.00-3079.38)	18.76(8.96-28.56)

	**Female**	461.88(258.25-665.51)	23.08(13.35-32.82)	375.49(115.53-635.45)	16.24(3.85-28.63)
	**Age**				
	15 to 19	91.16(35.34-146.99)	15.19(1.20-29.18)	68.21(6.17-130.26)	15.12(0.00-31.89)
	20 to 24	146.54(77.58-215.50)	44.10(26.53-61.66)	117.30(38.99-195.62)	31.81(10.08-53.53)
	25 to 29	189.66(119.01-260.31)	54.60(35.89-73.31)	160.71(69.73-251.68)	46.23(21.99-70.47)
	30 to 34	182.91(66.08-299.73)	43.62(28.43-58.82)	170.64(30.65-310.63)	38.89(18.95-58.83)
	35 to 39	96.54(53.93-139.14)	33.40(19.38-47.42)	102.12(47.35-156.88)	29.64(11.88-47.41)
	40 to 44	64.89(36.95-92.83)	23.70(14.91-32.48)	57.09(21.92-92.25)	18.26(7.07-29.44)
	45 to 49	42.06(22.00-62.12)	17.73(9.39-26.07)	36.96(11.98-61.94)	12.85(2.86-22.84)
	50 to 54	12.57(0.00-42.55)	13.98(7.51-20.45)	0.25(0.00-76.96)	10.57(2.99-18.14)
	55 to 59	18.99(0.00-39.31)	2.96(0.00-20.92)	12.40(0.00-64.38)	0.00(0.00-45.09)
	60 to 64	15.32(2.60-28.05)	6.36(0.00-13.96)	11.11(0.00-43.66)	4.35(0.00-23.79)
	65 to 69	16.62(11.97-21.28)	2.02(0.00-11.61)	19.11(13.24-24.97)	0.00(0.00-23.30)
**G20**	70 to 74	5.62(0.00-17.99)	4.92(0.09-9.74)	5.69(0.00-39.41)	5.62(0.51-10.74)
	75 to 79	2.71(0.00-9.02)	5.63(1.90-9.36)	2.71(0.00-11.71)	4.15(0.08-8.22)
	**Regions**				
	Argentina	14.00(12.28-15.72)	29.66(26.28-33.04)	16.21(13.81-18.62)	33.18(28.52-37.85)
	Australia	1.49(0.97-2.01)	6.05(3.33-8.78)	1.49(0.77-2.21)	6.05(2.28-9.82)
	Brazil	65.90(34.25-97.56)	29.96(13.94-45.98)	65.90(19.50-112.31)	29.96(6.79-53.14)
	Canada	3.35(0.00-7.34)	8.07(4.87-11.26)	3.38(0.00-13.39)	8.16(4.95-11.37)
	China	29.59(0.00-101.03)	1.99(0.00-7.51)	27.52(0.00-210.25)	1.76(0.00-15.89)
	European Union	22.24(17.03-27.45)	4.53(3.48-5.59)	22.57(17.23-27.92)	4.48(3.39-5.57)
	France	2.56(0.24-4.89)	3.86(0.00-7.95)	2.56(0.00-5.93)	3.86(0.00-9.81)
	Germany	2.31(1.16-3.47)	2.72(1.32-4.13)	2.31(0.71-3.91)	2.72(0.78-4.67)
	India	71.29(0.00-317.61)	5.47(0.00-28.69)	60.09(0.00-349.99)	4.29(0.00-31.04)
	Indonesia	14.22(5.56-22.87)	5.42(1.88-8.95)	14.22(1.32-27.11)	5.42(0.23-10.60)

Italy	3.07(0.73-5.40)	5.11(0.97-9.26)	2.99(0.62-5.35)	5.02(0.79-9.25)
Japan	3.49(2.49-4.49)	2.73(1.97-3.49)	4.20(2.82-5.58)	3.28(2.24-4.33)
Mexico	18.53(0.00-39.31)	11.88(4.88-18.89)	20.02(0.00-73.17)	11.44(4.03-18.85)
Republic of Korea	1.27(0.00-2.85)	2.61(0.00-5.93)	1.27(0.00-2.85)	2.62(0.00-5.95)
Russian Federation	106.85(3.69-210.01)	72.67(2.25-143.09)	105.77(0.00-277.26)	71.89(0.00-189.23)
Saudi Arabia	1.33(1.02-1.65)	2.94(1.25-4.64)	1.61(1.14-2.08)	2.95(0.12-5.77)
South Africa	486.13(0.00-1004.93)	705.26(0.00-2081.05)	398.57(0.00-1021.83)	349.92(0.00-2642.45)
Turkey	0.60(0.47-0.72)	0.73(0.55-0.92)	0.74(0.57-0.91)	0.90(0.64-1.15)
United Kingdom	5.51(1.42-9.59)	8.08(1.24-14.92)	5.51(0.00-11.91)	8.08(0.00-18.86)
United States of America	67.13(32.75-101.52)	17.84(12.06-23.62)	67.13(19.62-114.65)	17.73(11.94-23.51)

Table S8. The prediction of mortality in next 20 years in China and G20

Characteristics		2030		2040	
		**Mortality cases**	**ASMR per 100,000**	**Mortality cases**	**ASMR per 100,000**
		**No.×10^3 (95% UI)**	**No. (95% UI)**	**No.×10^3 (95% UI)**	**No. (95% UI)**
**China**	**Total**	41.07(29.69-52.45)	2.88(2.07-3.68)	50.72(34.61-66.84)	3.54(2.40-4.68)
	**Male**	30.51(21.47-39.56)	4.24(3.09-5.40)	37.71(24.73-50.69)	5.23(3.60-6.87)
	**Female**	10.30(6.83-13.77)	1.47(0.97-1.97)	12.66(7.65-17.67)	1.80(1.08-2.52)
	**Age**				
	15 to 19	0.22(0.09-0.34)	0.39(0.27-0.50)	0.22(0.00-0.44)	0.51(0.22-0.80)
	20 to 24	0.56(0.16-0.96)	0.94(0.74-1.14)	0.56(0.00-1.22)	1.15(0.85-1.45)
	25 to 29	2.23(1.39-3.06)	2.17(1.83-2.51)	2.72(1.53-3.90)	2.64(2.17-3.12)
	30 to 34	4.57(3.07-6.08)	3.41(2.64-4.18)	5.64(3.44-7.83)	4.18(3.10-5.26)
	35 to 39	4.22(3.07-5.37)	4.13(3.24-5.02)	5.20(3.58-6.81)	5.08(3.83-6.33)
	40 to 44	4.49(3.13-5.85)	4.29(3.12-5.46)	5.55(3.68-7.41)	5.26(3.65-6.88)
	45 to 49	5.66(4.14-7.18)	4.62(3.49-5.76)	7.02(4.90-9.13)	5.69(4.10-7.28)
	50 to 54	3.82(0.63-7.01)	4.11(2.66-5.57)	3.82(0.00-8.72)	5.07(3.01-7.13)
	55 to 59	4.79(2.71-6.88)	3.19(2.23-4.14)	7.05(1.93-12.18)	3.90(2.56-5.25)
	60 to 64	2.68(1.62-3.74)	3.25(2.03-4.47)	3.36(1.92-4.79)	3.98(2.26-5.70)
	65 to 69	2.46(0.00-6.54)	3.16(0.43-5.88)	2.41(0.00-13.00)	3.16(0.00-7.25)
	70 to 74	3.81(1.15-6.46)	4.75(3.06-6.45)	5.71(0.00-12.69)	5.87(3.45-8.28)
	75 to 79	1.23(0.86-1.60)	3.95(2.46-5.43)	1.52(1.00-2.04)	4.79(2.70-6.88)
	**Total**	216.50(0.00-805.85)	4.06(0.00-17.73)	157.99(0.00-1708.97)	2.50(0.00-38.47)
	**Male**	120.41(0.00-491.84)	4.49(0.00-21.97)	86.26(0.00-1063.66)	2.70(0.00-48.71)

	**Female**	96.19(0.00-351.12)	3.62(0.00-15.22)	71.92(0.00-742.83)	2.29(0.00-32.81)
	**Age**				
	15 to 19	6.54(0.00-16.02)	1.87(0.56-3.18)	7.30(0.00-32.39)	1.86(0.00-3.83)
	20 to 24	9.55(0.00-40.78)	4.37(1.44-7.30)	10.07(0.00-98.19)	4.26(0.94-7.58)
	25 to 29	8.58(0.00-72.05)	6.17(0.00-31.11)	-3.21(0.00-163.10)	7.12(0.00-77.17)
	30 to 34	25.10(0.00-135.41)	4.61(0.00-36.84)	13.12(0.00-301.72)	0.00(0.00-84.16)
	35 to 39	34.33(0.00-162.24)	7.65(0.00-47.79)	25.06(0.00-360.63)	3.13(0.00-108.43)
	40 to 44	34.91(0.00-157.96)	8.25(0.00-45.23)	30.71(0.00-363.71)	4.55(0.00-101.84)
	45 to 49	36.72(0.00-117.86)	8.55(0.00-40.39)	37.29(0.00-246.45)	6.62(0.00-90.31)
	50 to 54	24.17(0.00-72.24)	6.56(0.00-25.85)	20.90(0.00-147.15)	4.60(0.00-55.18)
	55 to 59	19.83(0.00-45.47)	3.80(0.00-19.55)	21.66(0.00-89.03)	1.34(0.00-43.69)
	60 to 64	15.07(10.05-20.08)	4.16(0.00-15.23)	18.43(11.19-25.67)	3.43(0.00-33.49)
	65 to 69	10.61(6.90-14.33)	3.12(0.00-8.81)	13.00(7.79-18.21)	2.20(0.00-16.76)
**G20**	70 to 74	4.93(1.41-8.45)	2.55(0.00-7.80)	4.89(0.00-10.25)	2.29(0.00-16.06)
	75 to 79	2.11(0.09-4.12)	1.50(0.00-6.77)	2.10(0.00-5.44)	0.84(0.00-14.69)
	**Regions**				
	Argentina	1.57(0.00-6.51)	3.96(1.90-6.03)	1.38(0.00-14.01)	3.95(1.85-6.04)
	Australia	0.07(0.00-0.42)	0.29(0.00-2.23)	0.07(0.00-0.55)	0.29(0.00-2.97)
	Brazil	15.01(9.84-20.17)	7.96(5.14-10.77)	14.52(9.02-20.03)	8.08(5.13-11.02)
	Canada	0.25(0.00-1.62)	0.69(0.00-5.36)	0.25(0.00-2.16)	0.69(0.00-7.23)
	China	41.07(29.69-52.45)	2.88(2.07-3.68)	50.72(34.61-66.84)	3.54(2.40-4.68)
	European Union	3.80(0.00-20.06)	0.74(0.00-4.10)	3.80(0.00-26.63)	0.74(0.00-5.45)
	France	0.44(0.00-5.82)	0.66(0.00-9.84)	0.44(0.00-8.06)	0.66(0.00-13.66)
	Germany	0.41(0.00-2.60)	0.48(0.00-3.18)	0.41(0.00-3.50)	0.48(0.00-4.28)
	India	157.22(0.00-408.54)	11.69(0.00-33.47)	315.20(0.00-1130.52)	24.11(0.00-95.66)
	Indonesia	7.68(6.43-8.93)	2.95(2.45-3.44)	9.57(7.79-11.34)	3.67(2.96-4.38)

Italy	0.61(0.00-5.42)	1.02(0.00-9.53)	0.61(0.00-7.34)	1.02(0.00-12.91)
Japan	0.16(0.03-0.29)	0.13(0.03-0.22)	0.16(0.00-0.34)	0.13(0.00-0.26)
Mexico	4.41(0.00-8.92)	3.91(1.68-6.15)	3.85(0.00-14.72)	3.97(1.66-6.29)
Republic of Korea	0.14(0.06-0.21)	0.26(0.10-0.42)	0.14(0.03-0.24)	0.26(0.04-0.48)
Russian Federation	24.49(17.91-31.06)	16.71(12.24-21.19)	29.76(20.68-38.84)	20.33(14.16-26.51)
Saudi Arabia	0.73(0.59-0.87)	1.42(0.60-2.23)	0.76(0.43-1.10)	0.90(0.00-3.25)
South Africa	325.11(0.00-846.49)	554.97(0.00-1546.84)	497.60(0.00-1916.16)	837.96(0.00-3535.52)
Turkey	0.31(0.22-0.40)	0.38(0.26-0.50)	0.39(0.27-0.51)	0.47(0.31-0.63)
United Kingdom	0.23(0.00-1.03)	0.34(0.00-1.74)	0.23(0.00-1.39)	0.34(0.00-2.36)
United States of America	0.00(0.00-14.81)	0.00(0.00-4.92)	0.00(0.00-10.45)	0.00(0.00-3.06)

Table S9. The prediction of incidence in next 20 years worldwide

Characteristics	2030		2040	
	Incidence cases No.×10^3 (95% UI)	ASIR per 100,000 No. (95% UI)	Incidence cases No.×10^3 (95% UI)	ASIR per 100,000 No. (95% UI)
**Total**	2565.89(0.00-5273.71)	35.10(0.00-83.36)	3646.66(0.00-12544.83)	57.13(0.00-227.61)
**Male**	1036.42(0.00-2251.25)	25.99(0.00-69.59)	1156.33(0.00-4811.98)	29.82(0.00-166.09)
**Female**	1572.36(88.91-3055.82)	45.54(0.00-95.98)	2704.71(0.00-7923.11)	93.78(0.00-288.67)
**Age**				
15 to 19	220.19(0.00-520.48)	34.59(0.00-94.54)	321.04(0.00-1397.30)	53.11(0.00-278.02)
20 to 24	264.01(0.00-694.29)	42.89(0.00-129.74)	318.69(0.00-1615.10)	51.03(0.00-314.66)
25 to 29	490.12(0.00-1099.26)	91.69(0.00-207.33)	669.01(0.00-2671.27)	134.74(0.00-520.14)
30 to 34	402.01(96.66-707.36)	71.29(0.00-149.84)	394.99(0.00-883.02)	70.07(0.00-196.72)
35 to 39	257.39(24.11-490.66)	47.10(0.00-103.99)	261.00(0.00-688.40)	47.48(0.00-133.96)
40 to 44	145.40(0.00-332.58)	28.07(0.00-56.59)	177.86(0.00-818.16)	31.63(0.00-80.90)
45 to 49	82.17(0.00-167.73)	20.78(0.00-61.77)	76.22(0.00-300.95)	21.53(0.00-95.83)
50 to 54	65.64(3.01-128.26)	13.76(0.00-45.53)	72.40(0.00-242.63)	14.26(0.00-100.68)
55 to 59	48.52(12.92-84.13)	6.29(0.00-24.30)	45.48(0.00-138.66)	0.00(0.00-47.30)
60 to 64	26.32(9.83-42.82)	11.50(0.00-25.82)	26.29(0.00-66.30)	12.02(0.00-58.03)
65 to 69	16.80(12.42-21.19)	5.20(0.00-16.16)	23.48(17.64-29.32)	1.36(0.00-30.67)
70 to 74	11.78(0.00-27.23)	3.26(0.00-20.95)	12.67(0.00-55.30)	1.33(0.00-53.14)
75 to 79	6.98(3.83-10.12)	2.72(0.00-10.06)	5.58(2.13-9.03)	2.70(0.00-14.45)
**SDI regions**				
Low SDI	1287.67(365.43-2209.90)	116.43(0.00-257.10)	1457.82(0.00-3128.43)	96.41(0.00-350.12)

Low-middle SDI	813.14(0.00-1727.52)	42.46(0.00-98.28)	1655.62(0.00-5389.49)	54.61(0.00-194.26)
Middle SDI	631.05(140.14-1121.96)	32.82(10.07-55.57)	697.01(157.31-1236.70)	35.01(9.59-60.44)
High-middle SDI	230.17(84.16-376.17)	16.04(5.64-26.45)	230.08(14.05-446.12)	16.03(0.83-31.24)
High SDI	94.03(58.53-129.54)	8.29(6.37-10.21)	94.03(44.98-143.09)	8.23(6.31-10.15)

Table S10. The prediction of mortality in next 20 years worldwide

Characteristics	2030		2040	
	Mortality cases	ASMR per 100,000	Mortality cases	ASMR per 100,000
	No.×10^3 (95% UI)	No. (95% UI)	No.×10^3 (95% UI)	No. (95% UI)
**Total**	840.11(279.19-1401.04)	11.19(2.40-19.98)	822.76(115.93-1529.59)	11.21(0.25-22.18)
**Male**	368.33(0.00-1030.98)	14.58(6.01-23.14)	308.51(0.00-2086.75)	18.48(8.72-28.23)
**Female**	657.37(383.46-931.29)	20.08(11.76-28.39)	717.76(411.13-1024.39)	23.16(13.67-32.65)
**Age**				
15 to 19	28.93(8.23-49.63)	4.41(0.90-7.93)	30.08(0.00-84.59)	4.36(0.00-13.62)
20 to 24	24.34(0.00-65.21)	8.23(0.00-22.62)	6.68(0.00-86.76)	11.47(0.00-55.40)
25 to 29	65.85(2.15-129.56)	25.06(14.03-36.09)	67.04(0.00-146.36)	27.86(15.16-40.56)
30 to 34	165.06(84.58-245.54)	31.97(15.85-48.08)	193.18(101.17-285.19)	40.95(22.43-59.46)
35 to 39	83.44(0.00-276.62)	23.24(4.10-42.38)	45.11(0.00-554.30)	23.25(0.00-47.21)
40 to 44	124.25(0.00-351.97)	36.41(12.76-60.07)	125.42(0.00-736.52)	39.93(13.35-66.51)
45 to 49	117.40(0.00-313.97)	34.17(14.55-53.79)	132.80(0.00-696.99)	35.85(13.91-57.80)
50 to 54	84.03(0.00-206.30)	24.72(9.28-40.16)	88.59(0.00-425.81)	26.63(9.23-44.03)
55 to 59	56.84(3.67-110.01)	13.16(4.10-22.23)	63.08(0.00-208.32)	12.85(1.40-24.31)
60 to 64	32.91(0.00-68.77)	12.42(4.39-20.45)	35.02(0.00-135.40)	12.58(3.64-21.52)
65 to 69	20.38(1.63-39.14)	8.81(3.97-13.64)	21.25(0.00-70.67)	10.00(4.53-15.47)
70 to 74	16.10(1.02-31.17)	7.38(3.58-11.18)	22.29(0.00-65.21)	7.65(3.33-11.96)
75 to 79	5.06(0.00-11.28)	3.84(0.92-6.76)	5.19(0.00-21.56)	3.83(0.23-7.43)
**SDI regions**				
Low SDI	232.63(0.00-617.98)	42.30(0.00-121.90)	112.76(0.00-846.96)	61.43(0.00-284.33)

Low-middle SDI	245.36(58.12-432.59)	14.32(0.87-27.78)	237.73(1.79-473.67)	14.20(0.00-30.97)
Middle SDI	249.16(0.00-682.63)	11.66(0.00-39.56)	222.91(0.00-1363.14)	13.20(0.00-95.41)
High-middle SDI	30.24(0.00-92.30)	2.27(0.00-11.55)	11.55(0.00-160.57)	1.10(0.00-24.85)
High SDI	10.32(0.00-45.55)	1.02(0.00-5.31)	10.34(0.00-57.06)	1.02(0.00-6.72)

Table S11. The prediction of DALY in next 20 years worldwide

Characteristics	2030		2040	
	DALY cases No.×10^3 (95% UI)	ASDR per 100,000 No. (95% UI)	DALY cases No.×10^3 (95% UI)	ASDR per 100,000 No. (95% UI)
**Total**	46646.56(15113.37-78179.75)	455.51(0.00-1335.18)	45928.78(6255.27-85602.28)	210.90(0.00-1866.90)
**Male**	26919.44(10990.62-42848.26)	605.09(186.11-1024.07)	32056.46(13631.17-50481.75)	607.61(84.99-1130.23)
**Female**	35900.09(19467.05-52333.14)	1166.42(713.27-1619.56)	41672.02(22521.45-60822.59)	1371.67(850.03-1893.31)
**Age**				
15 to 19	2111.89(644.94-3578.83)	321.37(70.45-572.29)	2124.20(0.00-5987.76)	305.63(0.00-967.29)
20 to 24	2572.79(777.87-4367.72)	577.66(0.00-1577.53)	2582.67(352.65-4812.69)	787.36(0.00-3858.33)
25 to 29	4454.62(410.01-8499.24)	1636.07(900.48-2371.65)	4514.17(0.00-9553.93)	1781.39(934.27-2628.50)
30 to 34	4206.54(0.00-13118.54)	1855.05(905.94-2804.16)	1190.78(0.00-18628.89)	2419.55(1325.26-3513.85)
35 to 39	4883.48(0.00-14998.85)	1975.80(932.55-3019.05)	2839.16(0.00-29491.31)	2449.72(1263.02-3636.42)
40 to 44	6252.77(0.00-17047.27)	1798.00(787.29-2808.70)	6201.58(0.00-35156.21)	2034.81(892.27-3177.34)
45 to 49	5322.92(0.00-13760.83)	1392.96(521.21-2264.72)	5926.03(0.00-30138.37)	1568.27(586.30-2550.24)
50 to 54	3440.84(0.00-8081.91)	967.29(430.22-1504.35)	3611.87(0.00-16381.72)	1082.75(475.92-1689.58)
55 to 59	2115.85(365.49-3866.21)	551.31(215.25-887.38)	2365.33(0.00-7120.38)	653.15(272.84-1033.46)
60 to 64	1110.20(84.11-2136.29)	375.57(138.46-612.69)	1217.97(0.00-4076.88)	375.74(111.48-640.00)
65 to 69	631.45(18.42-1244.48)	226.90(105.15-348.65)	718.82(0.00-2485.05)	250.39(112.57-388.21)
70 to 74	389.77(87.23-692.31)	150.21(61.01-239.41)	541.88(0.00-1401.46)	157.63(57.49-257.77)
75 to 79	106.76(7.75-205.77)	74.25(26.81-121.69)	116.29(0.00-376.93)	73.19(14.42-131.96)
**SDI regions**				
Low SDI	12057.33(0.00-33506.35)	1851.11(0.00-6542.58)	4662.60(0.00-46183.54)	2559.73(0.00-16427.80)

Low-middle SDI	13489.88(2903.57-24076.19)	1391.81(709.23-2074.40)	13155.00(0.00-26468.64)	1523.35(696.67-2350.04)
Middle SDI	15371.75(0.00-45816.31)	649.26(0.00-2105.40)	17374.09(0.00-105410.16)	767.45(0.00-5065.55)
High-middle SDI	1778.77(0.00-5023.30)	133.77(0.00-607.26)	893.30(0.00-8713.87)	80.42(0.00-1291.62)
High SDI	602.21(0.00-2447.64)	59.62(0.00-284.55)	601.77(0.00-3054.69)	59.59(0.00-358.98)

Table S12. The prediction of MIR in next 20 years worldwide

Characteristics	2030	2040
	MIR	MIR
**Total**	0.50(0.00-1.18)	0.59(0.00-2.64)
**Male**	0.35(0.00-0.87)	0.27(0.00-1.64)
**Female**	0.54(0.00-1.22)	0.68(0.00-2.83)
**Age**		
15 to 19	0.20(0.15-0.24)	0.24(0.17-0.31)
20 to 24	0.16(0.00-0.35)	0.17(0.00-0.72)
25 to 29	0.23(0.09-0.38)	0.25(0.09-0.41)
30 to 34	0.46(0.15-0.76)	0.44(0.10-0.78)
35 to 39	0.61(0.00-1.90)	0.77(0.00-4.50)
40 to 44	0.93(0.00-2.76)	1.00(0.00-6.25)
45 to 49	1.35(0.00-3.28)	1.52(0.00-7.23)
50 to 54	1.30(0.00-2.79)	1.26(0.00-5.24)
55 to 59	1.17(0.08-2.26)	1.34(0.00-4.31)
60 to 64	0.80(0.00-2.08)	0.78(0.00-4.41)
65 to 69	1.00(0.05-1.95)	1.18(0.00-3.68)
70 to 74	1.01(0.49-1.52)	1.01(0.09-1.93)
75 to 79	0.50(0.13-0.86)	1.16(0.68-1.63)
**SDI regions**		
Low SDI	0.51(0.00-1.12)	0.52(0.00-2.11)
Low-middle SDI	0.55(0.00-1.38)	0.53(0.00-2.97)

Middle SDI	0.46(0.00-1.15)	0.52(0.00-2.51)
High-middle SDI	0.46(0.17-0.75)	0.39(0.08-0.70)
High SDI	0.11(0.00-1.21)	0.11(0.00-1.65)
